# Biomass waste-to-energy valorisation technologies: a review case for banana processing in Uganda

**DOI:** 10.1186/s13068-016-0689-5

**Published:** 2017-01-03

**Authors:** Robert Gumisiriza, Joseph Funa Hawumba, Mackay Okure, Oliver Hensel

**Affiliations:** 1 School of Biosciences, Makerere University, P.O Box 7062, Kampala, Uganda; 2 School of Engineering, Makerere University, P.O Box 7062, Kampala, Uganda; 3 Universität Kassel-FG Agrartechnik, Nordbahnhofstr.1a, 37213 Witzenhausen, Germany

**Keywords:** Banana waste, Waste-to-energy technologies, Biomass valorisation, Bioenergy, Biofuels, Biomass energy, Anaerobic digestion

## Abstract

**Background:**

Uganda’s banana industry is heavily impeded by the lack of cheap, reliable and sustainable energy mainly needed for processing of banana fruit into pulp and subsequent drying into chips before milling into banana flour that has several uses in the bakery industry, among others. Uganda has one of the lowest electricity access levels, estimated at only 2–3% in rural areas where most of the banana growing is located. In addition, most banana farmers have limited financial capacity to access modern solar energy technologies that can generate sufficient energy for industrial processing. Besides energy scarcity and unreliability, banana production, marketing and industrial processing generate large quantities of organic wastes that are disposed of majorly by unregulated dumping in places such as swamps, thereby forming huge putrefying biomass that emit green house gases (methane and carbon dioxide). On the other hand, the energy content of banana waste, if harnessed through appropriate waste-to-energy technologies, would not only solve the energy requirement for processing of banana pulp, but would also offer an additional benefit of avoiding fossil fuels through the use of renewable energy.

**Main body:**

The potential waste-to-energy technologies that can be used in valorisation of banana waste can be grouped into three: Thermal (Direct combustion and Incineration), Thermo-chemical (Torrefaction, Plasma treatment, Gasification and Pyrolysis) and Biochemical (Composting, Ethanol fermentation and Anaerobic Digestion). However, due to high moisture content of banana waste, direct application of either thermal or thermo-chemical waste-to-energy technologies is challenging. Although, supercritical water gasification does not require drying of feedstock beforehand and can be a promising thermo-chemical technology for gasification of wet biomass such as banana waste, it is an expensive technology that may not be adopted by banana farmers in Uganda. Biochemical conversion technologies are reported to be more eco-friendly and appropriate for waste biomass with high moisture content such as banana waste.

**Conclusion:**

Uganda’s banana industrialisation is rural based with limited technical knowledge and economic capability to setup modern solar technologies and thermo-conversions for drying banana fruit pulp. This review explored the advantages of various waste-to-energy technologies as well as their shortfalls. Anaerobic digestion stands out as the most feasible and appropriate waste-to-energy technology for solving the energy scarcity and waste burden in banana industry. Finally, potential options for the enhancement of anaerobic digestion of banana waste were also elucidated.

## Background

Globally, energy crisis and proper waste disposal are among the major challenges facing most nations [5]. Uganda is the second largest global producer of bananas after India and the leading in Africa [164], with annual production estimated at 9.77 million tonnes [59]. The most widely grown cultivars are cooking types belonging to the East African highland banana (EAHB) subgroup. The other banana cultivars grown in Uganda include the dessert bananas locally known as *Sukali Ndizi* and *Bogoya* and some other plantain cultivars for roasting such as *Gonja* and *Kivuuvu* while ‘*Kayinja*’ and ‘*Kisubi*’ are mainly for making local beer. The EAHB cooking banana (AAA-EA group), locally called *matooke,* is the leading staple food [166] with the annual production of over 6 million tonnes [155]. Banana growing in Uganda is either cultivation by smallholders in association with other food crops at low densities (as shade trees for perennials such as coffee) or in commercial plantations at high densities in a monoculture system.

Banana processing in Uganda, like other agro-processing, relies mainly on costly imported petroleum products for energy. Cheap and sustainable energy is critically essential in banana processing for efficient drying of banana fruit pulp into chips prior to processing into value-added products such as starch and flour for export as well as local food security. Scarlat et al. [145] pointed out that access to cheap, reliable and sustainable energy is an important factor that makes agricultural and industrial processes more efficient. For instance, in the processing of banana, energy would be required for processes such as: drying, milling and also in conversion of the flour into valuable products: starch, bread and cakes, among others. Besides, energy is needed in households’ utilities such as cooking, lighting and refrigeration. The biggest challenge facing banana industry is the fact that banana-growing areas, that are concentrated in the rural as well as the remote parts of the country, are not connected to the national electricity grid. This makes banana processing not only expensive but also rather incomplete as there are many wastages. Typically, electricity distribution in Uganda is one of the lowest in Africa; estimated at only 9–12% of the total Ugandan population [99, 162] and at only 2–3% in the rural areas [168]. This is complicated by the fact that most banana farmers have limited financial capacity to access modern solar energy technologies that would generate sufficient energy for industrial processing. Therefore, such limited and unreliable energy access translates into underutilisation of the banana crop, excessive wastage, as well as emission of large volumes of banana waste, leading to the underdevelopment of the banana industry. This, in turn, contributes to the limited employment opportunities and poverty that are the major impediments to economic growth [82].

As already pointed out from the foregoing, banana production and banana fruit processing are not only faced with energy scarcity and unreliability, but also they are accompanied by the generation of vast quantities of waste. Banana Waste (BW) comprises the following fractions: rotten/damaged fruits, peels, fruit-bunch-stem (stalks), leaves, fibres, pseudo-stem and rhizome [1]. These fractions of banana wastes are generated from both, banana production and fruit processing. The waste category generated from the former includes all the off-cuts such as pseudo-stem, leaves, fibres and rhizome that remain in the garden after harvesting fruit bunches, while the latter generates residues such as peels, fruit-bunch-stem (stalks) and rotten/damaged fruits. Uganda’s banana fruit processing alone is estimated to generate more than three million tonnes of banana waste annually [155, 166], which means that it is possible to think of the waste as a resource for waste-to-energy conversion. Nevertheless, banana waste is currently heaped to decompose in uncontrolled manner thereby emitting large volumes of Green House Gases (GHGs) especially methane and carbon dioxide that are major drivers of climate change. In addition, leachate from BW dump sites contains high biological oxygen demand and nutrients which if channelled into water bodies aggravate climate change through eutrophication [83]. Since the main problem of banana industrialisation in Uganda is dual comprising: lack of cheap sustainable energy coupled with the emission of large quantities of organic waste residues, yet the solution to these problems seems to lie in the ability to convert banana waste into valuable energy. The development of either new or the adaptation of existing waste-to-energy technologies would not only solve the energy needs of the banana industry, but would also eliminate the waste burden with its accompanying environmental pollution. This review explores the various waste-to-energy technologies and evaluates their suitability in the generation of energy for use in the banana processing industry.

### Current banana waste utilisation in Uganda

Banana waste comprises rejected fruits, peels, fruit bunch stems, leaves, pseudo-stems and fibres. The management of banana waste has been largely by cultural means such as: (a) direct use pseudo-stems, fibres and leaves to mulch the plantations; (b) banana peels, leaves and fruit-bunch stalk are composted for manure; and (c) banana peels, rejected fruit fingers are fed to animals. However, cultural methods of managing banana wastes have recently been discouraged due to association with the rapid spread of plant diseases like the devastating banana bacterial wilt. Applying banana waste from infected banana plants into banana fields as mulches or compost manure is one of the leading means of transmitting banana bacterial wilt [89, 167]. There have been efforts towards utilising of banana fibres in the production of such products as paper, rope, table mats and handbags [137, 112]. Even these efforts are not economically viable since such products have very short lifespan. Hence, utilisation of banana waste through energy conversions could be the most appropriate venture for Uganda’s banana industrialisation.

### Energy requirement for banana processing

Banana processing in Uganda starts with cutting of mature banana fruit bunches from the pseudo-stems in the plantation. Subsequently, the fruit is de-bunched to separate fruit fingers; the fingers are peeled to get the pulp; the pulp is sliced, and finally dried into banana chips. The banana chips serve as the raw material for industrial banana processing into value-added products such as starch and flour, for both export and local food security. The drying of banana fruit pulp into chips is the step that requires reliable energy in order to produce consistently standard quality products. Moreover, it has been established [86, 142] that the drying of banana pulp consumes more energy than that of other related fresh foods such as pineapples and potato. This is so because the activation energy (Ea) for the diffusion of water in green banana is 51.21 kJ/mol which is higher than that for potato (32.24 kJ/mol), pineapple (35.17 kJ/mol) and grape seeds (30.45 kJ/mol) [85, 86, 142, 170]. The differences in the activation energy values can be attributed to the differences in the chemical composition and cellular structure [86]. In Uganda, the drying of banana pulp is done by directly spreading fresh banana fruit pulp on the mat and exposed directly to sunshine. Nevertheless, although Uganda is located on the equator, the number of hours of sunshine per day varies significantly depending on the season. During rainy season, there are few hours of sunshine that make the traditional drying method take many days resulting in the pulp either rotting, or infested with moulds that produce aflatoxins. Aflatoxin contamination is one of the major hindrances to the development of the banana industry as the products thereof would not meet the minimum standards for human consumption. Therefore direct sunshine drying, as done locally, does not meet the energy requirements for efficient and safe drying of the pulp for subsequent processing. Other options would be: (a) the use of modern solar dryers. This, however, has not been massively adopted due to the high cost of installation and (b) hot air convection drying. This is one of the oldest methods that have been used to preserve agricultural products like banana [143] and relies on the flow of hot air over the sliced pulp. Its application is, however, hampered by the high energy of operation [6, 94, 101, 117]. Therefore the conversion of waste biomass to energy would offer a cheap and affordable alternative source of energy for drying the pulp by banana growers and processors.

### Waste valorisation: a concept

**Fig. 1:**
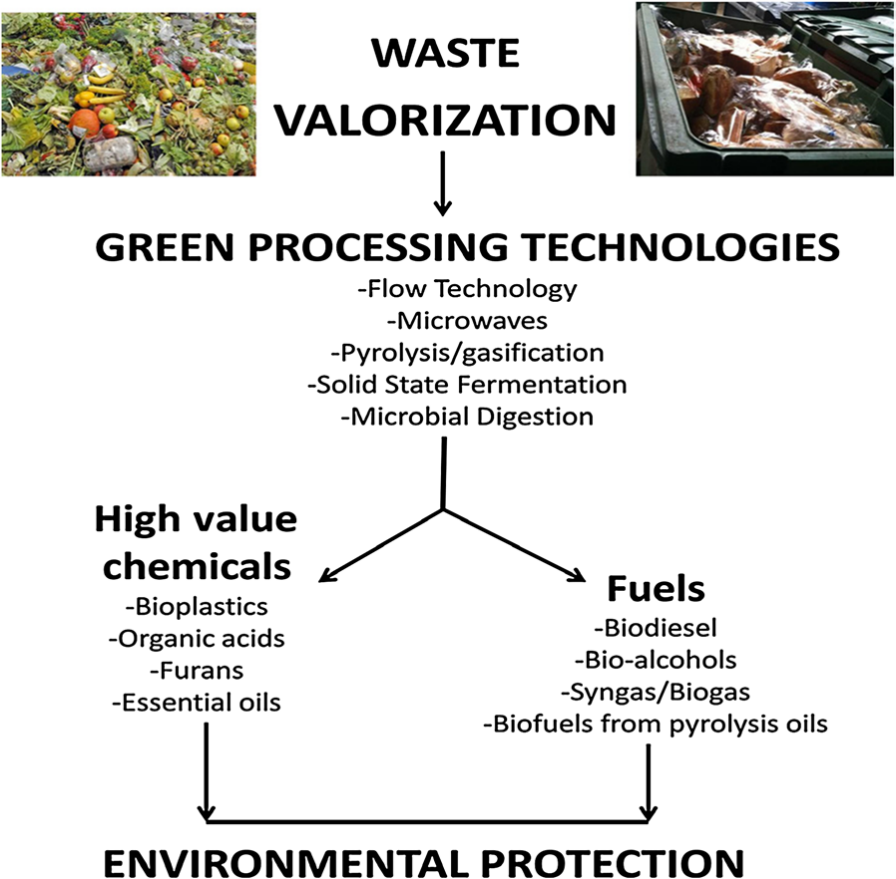
A scheme of green processing technologies for waste valorisation [13]

Waste valorisation has been defined as the process of converting waste materials into more useful products such as chemicals, materials and fuels [13]. Waste valorisation as a concept relies on the assumption that even after the intended use, the residue/waste still contains untapped polymeric substance that can be converted to either energy or other chemical forms. Such products make waste a valuable resource that should not be left unharnessed. This concept is currently being applied on both synthetic waste as well as biowaste, with promising success, and it is the basis of the current waste-to-energy (WtE) approaches. Moreover, due to the fast depletion of natural/primary resources, waste valorisation is not a luxury for academic exploration but rather a much needed technology for cost-effective and sustainable waste management options and generation of renewable energy as well as production of high-value chemicals such as ethanol and materials such as nano-bioplastics (Fig. 1). Apart from renewable energy and high-value chemicals, waste valorisation offers additional advantages including: amelioration of waste mal-odours and environmental pollution, and reduction of the volume of waste, resulting in the recovery of more space for other uses. In a typical process, high-value chemicals are produced from waste residues through any of the four downstream processing i.e. using inorganic and organic chemicals, a combination of chemicals and enzymes, biotechnological approach using genetically engineered organisms, and green processing technologies whereby only water is used as a reagent in waste volarisation [13].

Waste-to-Energy (WtE), defined as the process of recovering energy in the form of either electricity and/or heat from waste, [30] applies the waste valorisation concept to generate renewable energy such as heat and biofuels (biogas, syngas and bioethanol). Waste-to-Energy technologies are categorised into two major groups namely; (a) thermo-chemical processes comprising combustion, pyrolysis and gasification; and (b) biological processes comprising anaerobic digestion and bioethanol fermentation. These WtE technologies provide cheap sources of energy that is crucial for industrial processes such as drying, packaging and preservation industrial products. As already highlighted, the banana industry releases a large volume of waste that is currently neglected and left to decompose in an uncontrolled manner. Besides, the development of this industry is hampered by both scarcity and costly energy inputs. The application of this volarisation concept, particularly the green processing options, would solve both of these hindrances to the banana industrial development. Scarlat et al. [145] reported that the energy content of such wastes as banana waste can be recovered by employing appropriate WtE technologies. A number of studies have been conducted to establish the best way to harness energy from banana waste. For instance, banana wastes have been used to make briquettes that store energy for further uses in industrial and domestic heating [98, 149, 183]. In a separate study, Tock et al. [163] applied direct combustion of pseudo-stems and leaves to generate heat energy. The green processing option has been attempted [44, 163] whereby microorganisms have been employed to anaerobically convert banana peels into methane, in one study, and banana fruit residues fermented into ethanol [64, 74, 179] in another study. Thus, recovery of energy from waste can play a role in minimising the impact of waste on the environment with the additional benefit of providing a local source of cheap energy [145].

**Fig. 2:**
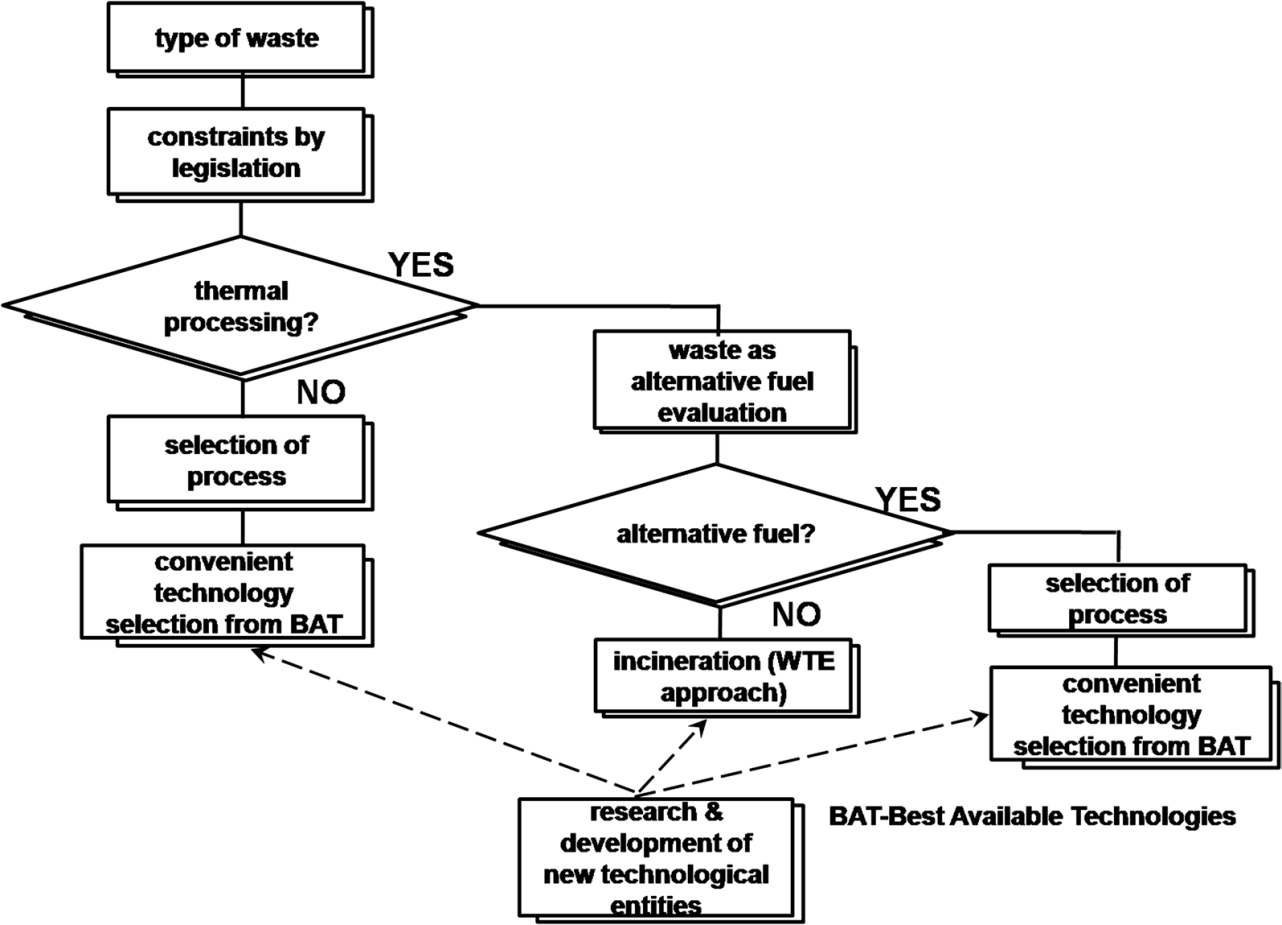
Algorithm for convenient WtE technology selection [156]

Development of innovative technologies with high WtE efficiencies is largely dependent on two major but inter-linked factors namely, the type of waste to be harnessed [174] and the available legislation. The legislation for environmental pollution abatement compels the waste sources (industries) to employ the most eco-friendly technologies for waste management. In addition, the physico-chemical nature of the waste dictates the choice of the technology appropriate for treating such a waste. As already mentioned in the foregoing, the WtE options are most preferred due to recovery of energy that can offset the cost of waste treatment. The energy content of waste is usually recovered by means of either thermo-chemical processes such as combustion, pyrolysis and gasification or biological processes such as anaerobic digestion. A possible algorithm (Fig. 2) for selecting or developing a suitable WtE technology has been described by Stehlik [156]. In this algorithm, the waste is first assessed for its suitability for thermal processing due to ease of application of thermal conversion technologies. Wastes that cannot be appropriately degraded by thermal means, the emitting industry either employs the existing non-thermal convenient technologies such as anaerobic digestion or supports research for development of new WtE technologies tailored to the type of waste emitted. On the other hand, wastes that are suited for degradation by thermal means are further evaluated for use as alternative fuels. Wastes that are not amenable for use as alternative fuel are degraded via incineration while for those that conform to use as alternative fuel are converted to energy via other WtE technologies such as pyrolysis, gasification as well as thermo-mechanical pulverisation to form refuse-derived fuel. Furthermore, the algorithm supports the need for research and development of new technologies in order to either improve on the efficiency of the available technologies and/or innovate new appropriate WtE technologies for waste management. These new technologies need to prove their economic viability prior to full-scale implementation. Generally, the simpler design has low propensity for technological failure.

## Potential WtE technologies for banana waste valorisation

**Fig. 3:**
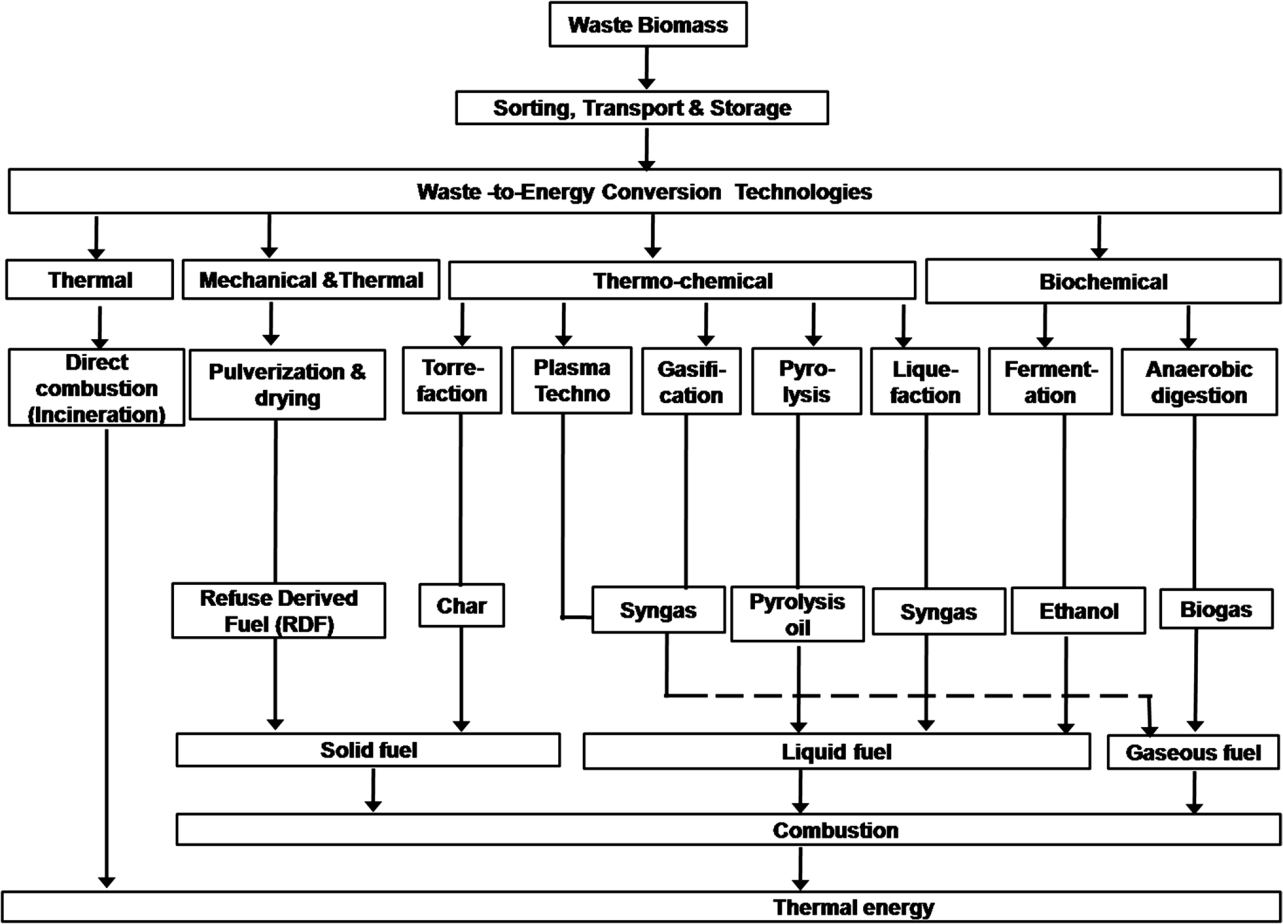
Potential WtE technologies for valorisation of banana waste [30]

The potential WtE technologies that can be used in the valorisation of BW can be grouped into: Thermal (Direct combustion and Incineration), Thermo-chemical (Torrefaction, Plasma treatment, Gasification and Pyrolysis) and Biochemical (Composting, Ethanol fermentation and Anaerobic Digestion) [30] Fig. 3. Generally, thermal technologies convert the waste directly into heat energy while thermo-chemical and biochemical ones first convert the waste into secondary energy carriers such as syngas, torrefied pellets, biogas, bioethanol and biooil, which can subsequently be burnt (in furnaces, steam turbine, gas turbine or gas engine) to produce energy in the form of heat and/or electricity. The conversion of solid wastes into secondary energy carriers allows for a cleaner and more efficient energy harnessing process.

### Thermal conversion technologies

This is the full oxidative combustion of waste biomass mainly to generate heat energy. This is done by either direct combustion or incineration. Direct combustion is the burning of biomass directly to convert chemical energy stored in plants into heat and electricity [45]. The direct burning of dry biomass to generate heat energy for mainly cooking and lighting has been practised globally for years. Dry banana waste such as leaves, fibres and fruit-bunch-stems can be used as a source of heat energy in domestic cooking and industrial boilers. Industrially, biomass is burnt in the furnace to generate thermal energy that subsequently heats boiler to produce steam. The pressure of the steam can be used to turn a turbine that is attached to an electrical generator which subsequently generates electricity [37]. The potential of banana residue to be directly combusted for energy generation strictly depends on its energy content or heating value [163]. However, banana residues have very high moisture content which lead to low net energy efficiency when combusted without prior drying process. Moreover, open burning of waste is particularly discouraged due to the emission of harmful compounds such as dioxins, acid gases and furans that cause air pollution [145]. Hence, direct combustion is not a suitable technology for harnessing energy from banana biomass.

**Fig. 4:**
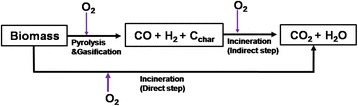
Key reaction steps and products from biomass combustion

Waste incineration, on the other hand, is a full oxidative combustion of the waste in an engineered structure called an incinerator with the purpose of generating thermal energy and simultaneous destruction of pathogenic waste material under emission control. During incineration, the biomass is converted either directly into CO_2_ and water vapour or indirectly into CO, H_2_ and Char (Fig. 4). The concentration of oxygen available for the process is the major determining factor. The direct step is favoured at higher oxygen concentrations while the latter occurs when there is limited oxygen supply. Waste incineration is common practice in the developed countries (EU, US, Japan) where waste-related policies limit waste disposal on land [145]. Although waste incineration appears simple and applicable for Uganda’s banana processing waste, the technology can be challenged by a number of bottlenecks. The high capital, maintenance and operation costs of waste incineration plants prevent the large-scale application of this technology as an energy recovery option [171, 172]. As with direct combustion, incineration is also affected by the high moisture content of banana waste, which makes continuous and optimal plant operation difficult to achieve owing to the requirement of additional fuel to support the process. Besides, without proper controls, waste incineration can be highly polluting, generating harmful emissions, such as dioxins and heavy metals.

### Thermo-chemical conversion technologies

**Fig. 5:**
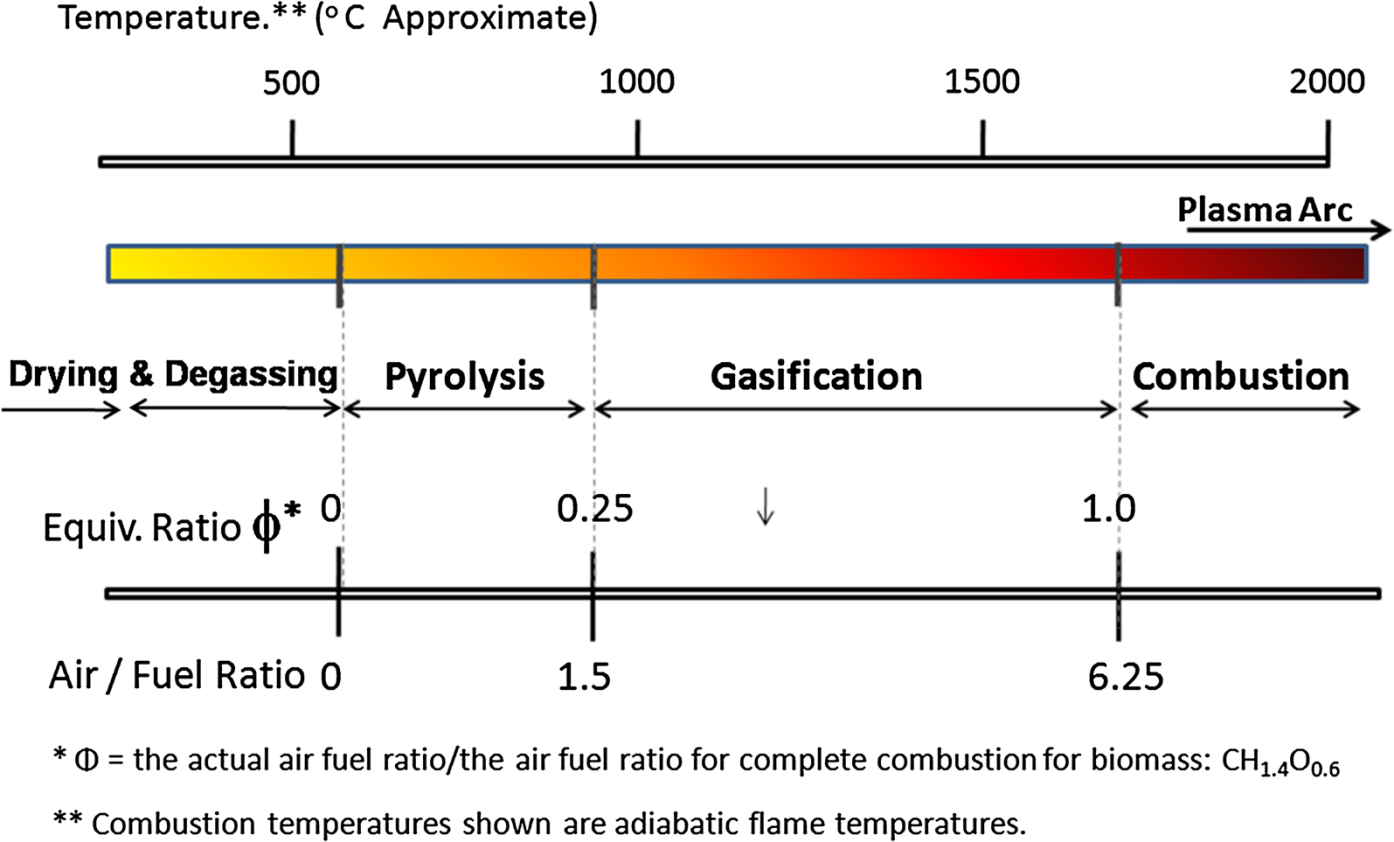
The temperature overlapping of thermo-chemical conversion technologies

Unlike incineration and open combustion, thermo-chemical conversion technologies employ a series of chemical reactions occurring at different temperatures and may require partial oxidation as in gasification or proceed in the absence of oxygen as in pyrolysis. These conversion technologies are temperature depended and proceed through overlapping spatial and temporal stages of drying and degassing, pyrolysis and gasification and finally full oxidative combustion that turns the organic waste into ash (Fig. 5). All these technologies require strict control of process conditions in specially designed reactors that are able to separate temperature accordingly. Without temperature separation and proper air rationing, thermo-chemical reactions do not occur ultimately, turning the process into incineration or combustion.

**Fig. 6:**
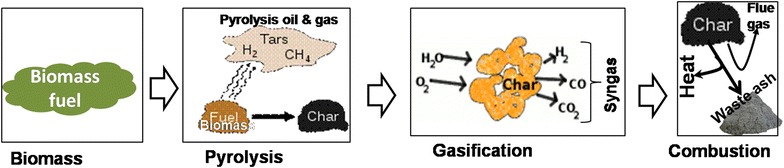
Sequential product generation during pyrolysis and gasification

Pyrolysis and gasification differ from incineration in that the former may be used for recovering the chemical value of the waste, while the latter is used to recover its energy value. The chemical products generated from pyrolysis and gasification may be either used as fuel to generate heat energy or as secondary feedstocks (char) for subsequent fuel generation (Fig. 6). The products from incineration are generally non-fuel and include ash and flue gas that mainly consists of carbon dioxide and water vapour.

Like incineration, pyrolysis and gasification also release carbon dioxide. A comparison of pyrolysis, gasification and combustion based on generated products is shown in Table 1. The principles underlying the application of each of the thermo-chemical conversion technologies in harnessing energy from biomass are here below described in detail:

**Table 1:**
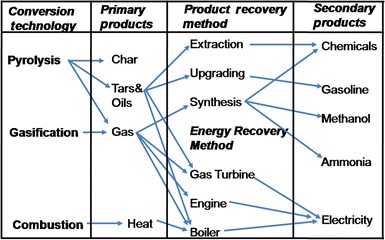
Thermo-conversion processes and products (Adapted from Bridgwater [32])

#### Pyrolysis

**Fig. 7:**
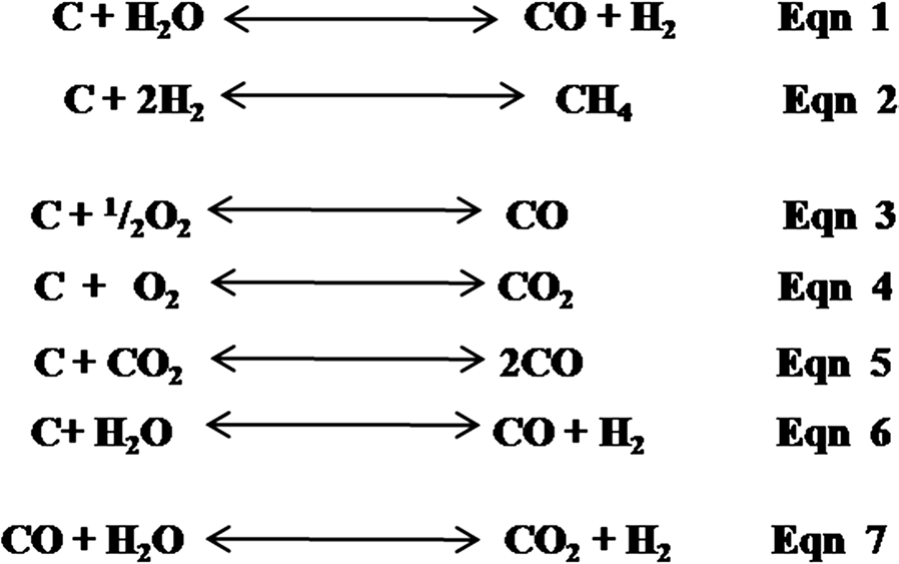
Major reactions of pyrolysis and gasification conversion technologies

Pyrolysis is the thermal degradation of organic material in the absence of oxygen. It occurs at relatively low temperatures (400–900 °C) [30]. In pyrolysis, biomass is subjected to an optimal temperature of 700 °C in the absence of oxygen resulting in the production of pyrolysis oil (biooil), char and synthesis gas (Syngas). Syngas is a mixture of majorly CO, CO_2_, H_2_, H_2_O, CH_4_, trace amounts of higher hydrocarbons such as ethane and propane, as well as various contaminants such as small char particles. These can be used as secondary fuel to generate electricity. In a typical process the biomass is transformed into high quality fuel without creating ash or emitting large volumes of flue gas as in combustion. The process proceeds through the following basic process stages: (1) grinding to increase the surface area for improved heat transfer and reaction; (2) drying to increase the efficiency of gas–solid reactions within the reactor; (3) anoxic thermal degradation of organics to generate pyrolysis products (pyrolysis gas, biooil and char); and (4) ultimate secondary treatment of pyrolysis gas and pyrolysis char. The last step involves the condensation of the gases for the extraction of energetically usable oil mixtures and/or combustion of gas and char as secondary energy products. The major gases generated from pyrolysis are methane, carbon monoxide and hydrogen and are shown by reaction Eqs. 1 and 2 (Fig. 7). Pyrolysis offers a flexible and attractive way of converting solid biomass into an easily stored and transportable fuel, which can be successfully used for the production of heat, power and chemicals. Pyrolysis gas, for example, may be used to power gas engines and gas turbines to generate electricity more efficiently than conventional steam boilers. Moreover, pyrolysis of biomass may lead to the recovery of organic liquid fraction as fuel in the form of methanol that can be distilled for use in various industries. Notably too, combustion of pyrolysis products emits smaller volumes of flue gas compared to direct combustion and incineration of biomass and hence pyrolysis reduces the flue gas treatment capital costs. Despite the advantages of pyrolysis, biomass with high ash content such as straw and banana waste are not good feedstocks for pyrolysis process due to reactor blockage by ash accumulation. Besides, pyrolysis is an expensive technology that requires high investment costs before it can be carried out commercially for energy harnessing.

#### Gasification

Gasification is a partial oxidation of organic substances at elevated temperature (500–1800 °C) to produce syngas. Biomass gasification occurs as the char reacts with carbon dioxide and water vapour (steam) to produce carbon monoxide and hydrogen via the reaction Eqs. 3–6 (Fig. 7). In addition, the concentrations of carbon monoxide, steam, carbon dioxide and hydrogen are balanced very fast at the temperatures in a gasifier via the equilibrium reaction Eq. 7 (Fig. 7). Syngas can be used as a fuel for efficient production of electricity and/or heat [169]. A gasifier can use oxygen, steam, carbon dioxide or a mixture of these as gasification agents.

On the other hand, banana waste being a wet biomass is not regarded as a promising feedstock for direct utilisation or application of the conventional thermo-chemical gasification processes due to its high moisture content [163]. This problem can be circumvented by employing a recently developed technology referred to as supercritical water gasification (SCWG) whereby water is used as a reaction medium. In this technology, gasification of wet biomass may be accomplished without having to dry the material and thereby avoiding the high processing costs associated with the drying process. Supercritical water gasification of wet biomass, as an advanced technology, has drawn the attention of a few research groups in the USA, Germany, Japan and the Netherlands [163]. The main advantage of using SCWG is that the technology does not require drying of wet biomass prior to gasification [62]. As a matter of fact, water in wet biomass is essential for the chemical reactions. Moreover, the SCWG of wet biomass results into high yields of hydrogen (H_2_) and very low yield of carbon monoxide (CO) when compared to the ‘‘dry processes’’ in which syngas is produced with CO as the main product. Besides, in SCWG less tar and coke are formed and inorganic ingredients such as salts remain in aqueous solution, thus corrosion problem during gas treatment can be avoided. Nevertheless, SCWG is an expensive technology which requires high capital investment before put into operation.

#### Plasma technology

Plasma technology relies on the physical principle that matter changes its state when energy is supplied to it: solids become liquid, and liquids become gaseous. When more energy is supplied to a gas, it is ionised and goes into the energy-rich plasma state, the fourth state of matter [126]. The initial energy required to create plasma can either be thermal or electric current or electromagnetic radiations. The presence of charged gaseous species makes the plasma highly reactive and causes it to behave significantly different from other gases, solids and liquids. The peculiar advantage of this technology is that the energy contained in the plasma allows the use of low energy biomass that would otherwise not be suitable as feedstock for energy generation using gasification technology. The high-temperature conditions that are reached in plasma results in the decomposition of organic compounds into their elemental constituents and ultimately forming a high-energy synthesis gas, constituted mainly of hydrogen and carbon monoxide. Nevertheless, the application of plasma-based systems for waste management is challenging. For instance, the use of electricity as an initial energy vector is expensive, turning economic considerations into the strongest barrier for using plasmas for waste treatment. Moreover, the inorganic fraction (glass, metals and silicates) that is melted and converted into a dense, inert, non-leaching vitrified slug can be hazardous when released to the environment.

#### Torrefaction

**Fig. 8:**
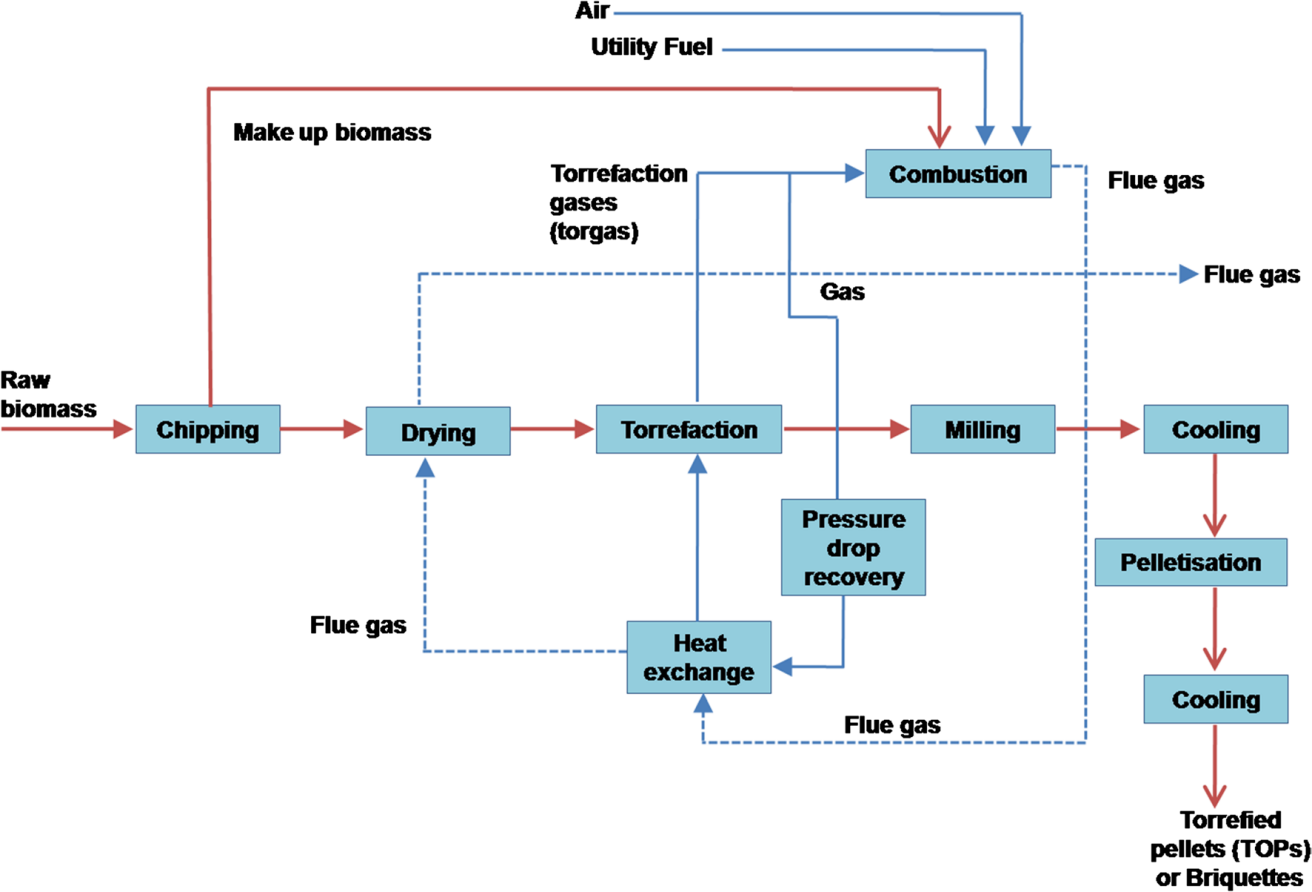
A flow scheme of an integrated torrefaction process based on [19]

Torrefaction is defined as the thermal upgrading of biomass into a more homogeneous product that is densified through pelletisation to generate a more energy-dense product called torrefied pellets (TOPs) or briquettes, with similar properties to coal [19]. The energy derived from biomass through thermal upgrading (heating) is concentrated into an energy-dense and homogeneous product (TOPs) useful for further thermo-chemical conversions [188]. Torrefaction technology is also referred to as mild pyrolysis and is a thermo-chemical process conducted in the temperature range between 200 and 300 °C under an inert atmosphere and low heating rate [110]. The process involves biomass chipping to allow efficient drying, screening for impurities before sizing [148] and drying to 20% moisture content (Fig. 8). A small fraction of the feedstock biomass is used as fuel for the drying and torrefaction process. Torrefied biomass (briquettes) which retains upto 96% of its chemical energy is hydrophobic and resistant to biodegradation. Therefore it can be used as substitute for coal/charcoal for domestic heating, co-firing power generation and gasification [3, 135, 139]. A study by Sellin et al. [149], in the Northern region of Santa Catarina in Brazil, revealed that banana wastes including leaves and pseudo-stems can be used to produce briquettes as fuel for energy generation. Briquettes produced from this waste at low cost are an excellent source of cheap renewable energy which is regarded as environmentally clean. Despite the potential of torrefaction technology, there are still several technical and economic challenges that need to be overcome before the technology is fully commercialised in the banana industry [127]. Firstly, banana waste like other plant biomass is highly heterogeneous in quality and nature, and is mostly available in low energy density form [50, 123, 181]. Secondly, it has relatively high moisture content and consequently lower heating value compared to fossil fuels [24, 40, 136]. It, therefore, needs to be pre-treated to improve handling [104, 131, 140]. Pre-treatment such as pre-drying to 20% moisture content is energy consuming and significantly reduce the energy efficiency of the technology.

### Biochemical conversion technologies

Biochemical conversion technologies of waste-to-energy are much more eco-friendly as compared to the thermal and thermo-chemical techniques discussed in the foregoing. The advantages and disadvantages of different waste-to-energy technologies are highlighted in Table 2. Biochemical conversion primarily involves the action of enzymes derived from microorganisms to harness the energy stored in biomass. The techniques falling under this category are: composting to generate heat energy, bioethanol fermentation and anaerobic digestion for biogas production.

**Table 2:** Advantages and disadvantages of different WtE technologies [90]

Technology	Advantages	Disadvantages
Anaerobic digestion	Energy recovery with the production of high grade soil conditioner	Unsuitable for wastes containing less organic matter
	No power requirement for sieving and turning of waste pile	Requires waste segregation for improving digestion efficiency
	Enclosed system enables trapping the gas produced for use	
	Controls GHG emissions	
	Free from bad odour, rodent and fly menace, visible pollution and social resistance	
	Compact design needs less land area	
	Net positive environmental gains	
	Can be done in small scale	
Landfill with gas recovery	Least cost option	Surface runoff during rainfall causes pollution
	Gas produced can be utilised for power generation or direct thermal application	Soil and groundwater may get polluted by the leachate
	Skilled personnel not required	Yields only 30–40% of the total gas generated
	Natural resources are returned to the soil and recycled	Large land area required
	Can convert marshy lands to useful areas	Significant transportation costs
		Cost of pre-treatment to upgrade the gas to pipeline quality and leachate treatment may be significant
		Spontaneous explosion due to methane gas buildup
Incineration	Most suitable for high calorific value waste	Least suited for aqueous, high moisture content, low calorific value and chlorinated waste
	Units with high throughput and continuous feed can be set up	Toxic metal concentration in ash, particulate emissions, SO_x_, NO_x_, chlorinated compounds, ranging from HCL to dioxins
	Thermal energy for power generation or direct heating	High capital and O&M costs
	Relatively noiseless and odourless	Skilled personnel required
	Low lands are required	
	Can be located within city limits, reducing transportation costs	
	Hygienic	
Pyrolysis/Gasification	Production of fuel gas/oil, which can be used for various purpose	Net energy recovery may suffer in waste with excessive moisture
	Control of pollution superior as compared to incineration	High viscosity of pyrolysis oil may be problematic for its burning and transportation

#### Composting

**Fig. 9:**
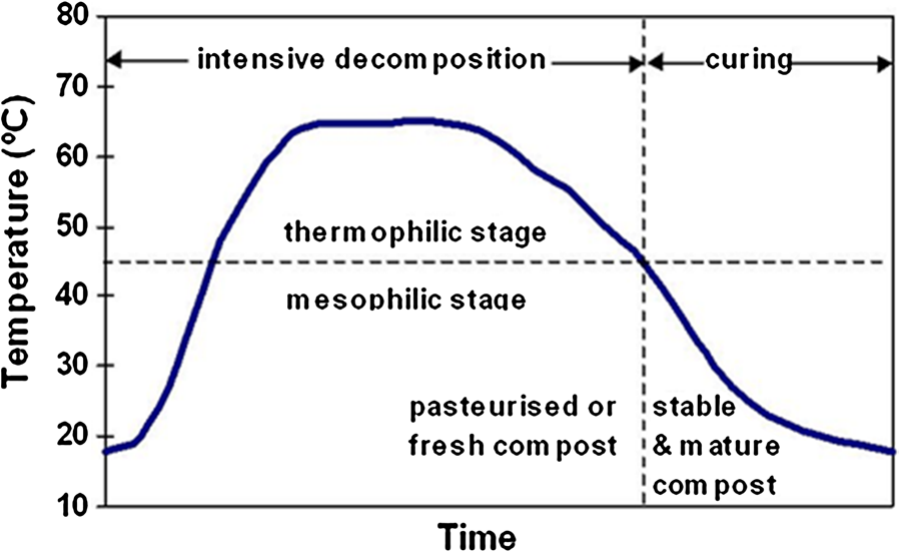
Heat generation during composting

Composting, defined as the biological decomposition of biodegradable solid waste under predominantly aerobic conditions, transforms the biomass into: carbon dioxide, water, heat and a more stable solid product called compost. The compost is nuisance-free, easy to handle and can be safely used in agriculture to ameliorate the soil [12, 84, 90]. Recently, there has been increased attention given to heat recovery from aerobic composting systems as a way to improve their economic viability [154]. Generally, the composting process is optimised by having the starting carbon to nitrogen ratio in the range of 30:1 and the moisture and oxygen levels and temperatures that are closely managed and monitored [58]. Three categories of microorganisms, namely, bacteria, actinomycetes and fungi are involved in the composting process. In the initial phase of composting, mesophilic microorganisms such as bacteria, *Bacillus, Clostridium, Alcaligenes, Serratia* and *Pseudomonas,* degrade biomass. This is accompanied by the generation of heat owing to their metabolic activities, causing the ensuing rise in temperature (≥45 °C) in the composting heap. This gives way to the second phase, whereby thermophiles take over the composting process. Thermophilic fungi such as *Aspergillus fumigates*, *Humicola* sp, *sporotrichum thermophile* and *Myriococcum thermophilum,* and *Streptomycetes thermofuscus*, *S. Rectus*, *Nocardia* sp and *Thermoactinomyces* sp continue with the process until the temperature of ≥50 °C is reached above which most of them are either inhibited or remain dormant as spores. Above 50 °C themophilic bacteria belonging to such genera as *Bacillus* (*Bacillus stearothermophilus*), *Thermus*, *Clostridium* continue with the process to temperatures ranging from 60 to as high as 65 °C (Fig. 9) and then starts to fall within a couple of months [152]. This sets in the third and final phase of the composting process. During this final stage, the actinomycetes, initially, followed later by fungi proceed with the composting process until the temperature falls to mesophilic range, after which both mesophilic fungi and bacteria re-colonise the compost heap to complete the process.

The mechanism of heat transfer has been described by Shaw and Stentiford [151]; Themeli [161] and Tucker [165] and involves convection and conduction, with radiation effects being assumed negligible. There are three components of energy balance namely; energy transfers into, within and out of a composting system which together equate to the change in energy stored within the system that ultimately dictates the temperature within the composting substrate. A study by Smith and Aber [154] reported an operational system capturing thermal energy in the hot air generated by the composting process, installed at the research farm of University of New Hampshire (UNH) in the United States.

**Fig. 10:**
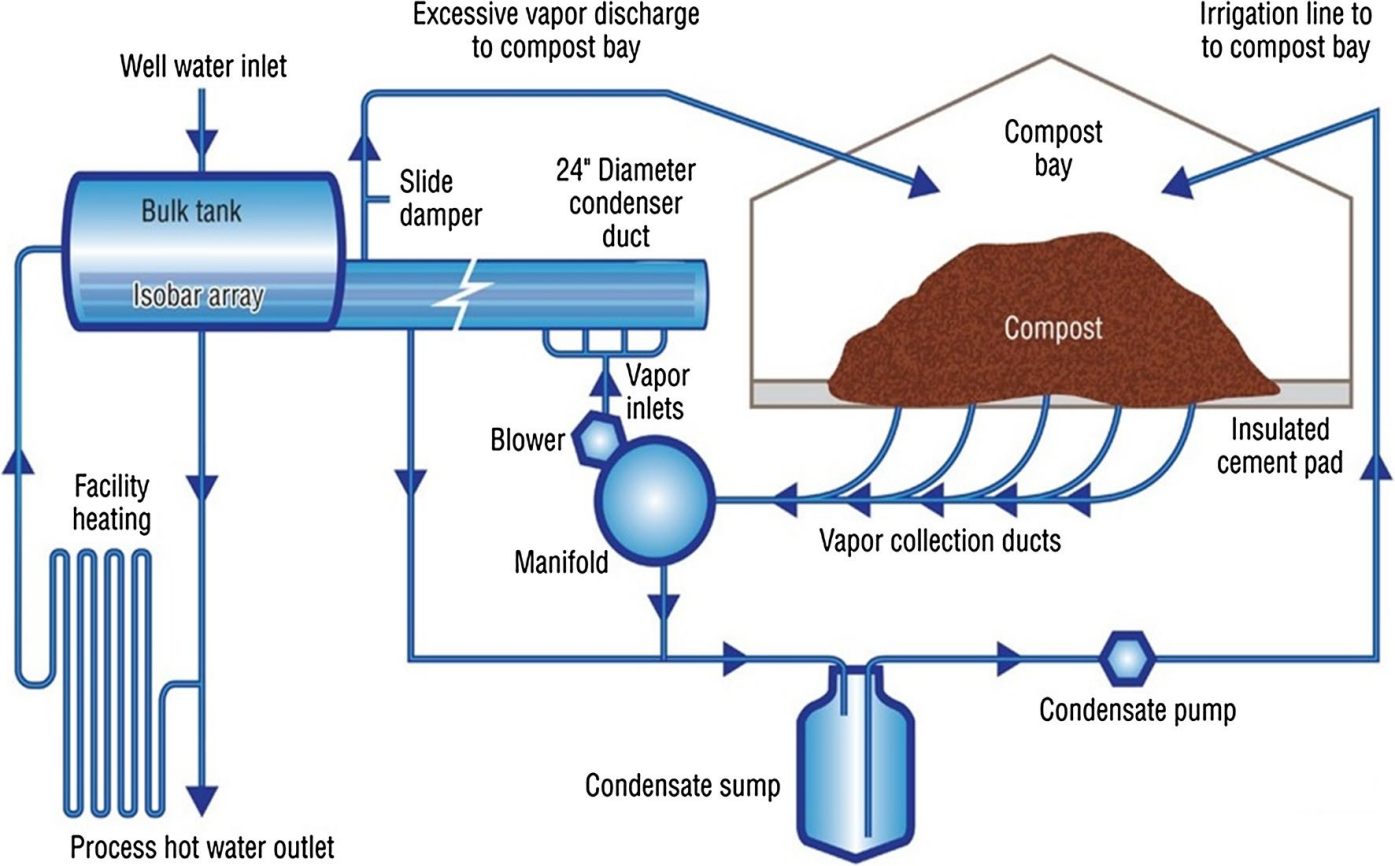
Flow diagram of UNH heat recovery system [154]

The system consists of an aerated static pile (ASP) of biomass or compost housed in a concrete insulated compost bay (Fig. 10). The hot vapour from the ASP is collected through PVC pipes that passes through manifold and connects to the heat exchange system. The condensate from the manifold and heat exchange system is collected through condensate sump and ultimately pumped back to the ASP in the compost bay. The heat exchange system operates by blowing hot compost vapour (110–170 °F), against an array of two-phase super-thermal conductor heat pipes termed as Isobars. These Isobars are 30 ft long containing within 24-in. diameter vapour duct and housed inside a 295-gallon water tank. Isobars provide thermal uniformity across the entire length of the pipe, thus heat energy is evenly distributed across the entire length of the pipe [2]. When compost heated vapour is applied to the evaporator side of the pipe (portion contained within the 24-in. diameter pipe), the refrigerant inside the Isobar heats up and vapourises. The vapour stream within the Isobar travels up the pipe, condensing on the cooler side, releasing its energy in the bulk storage water tank through the latent heat of condensation. After condensing, the refrigerant is returned to the warm end of the pipe through gravity, repeating the process without any moving parts.

The system captures the metabolic heat produced by microorganisms during aerobic composting, through a negatively aerated fan system, and blows the hot compost vapour (110–170 °F) against the heat exchange system to heat water for radiant floor heating, feed preparation and sanitation of equipment. However, the success in application of composting technology to generate thermal energy has been scantily reported elsewhere in the world. Moreover, composting of mixed wastes generates low quality compost which can introduce heavy metals into human food chain.

#### Bioethanol fermentation

Ethanol produced from different renewable feedstock constitutes an alternative fuel for spark ignition engines [179]. This ethanol is considered as biofuel due to the vegetative origin of its carbon and, therefore, when it is released during the combustion process, it will not contribute to the increase in CO_2_ emissions [76, 88]. The most suitable feedstock for ethanol production are high sugar-content crops such as sugarcane, sugar beets and fruits, since they majorly contain simple sugars such as glucose and fructose, that can be readily converted into ethanol by alcohol-fermenting microorganisms [56]. Two groups of microbes: saccharolytic and ethanologenic, are important in ethanol production. These groups operate on the principle of co-metabolism, whereby, when saccharolytic microbes break down complex polymeric carbohydrates (starch, cellulose, hemicelluloses, etc.) to simpler utilisable forms the ethanologenic converts them to ethanol. Many promising saccharolytic and ethanologenic microbes fall within, respectively, the phyla Neocallimastigomycota and Ascomycota, for fungi, Proteobacteria and Fibrobacteres, for bacteria. Notably, *Saccharomyces cerevisiae*. (Ascomycota) and *Zymomonas mobilis* (Proteobacteria) are the only microbes naturally capable of producing ethanol close to theoretical maximum, with *Saccharomyces cerevisiae* predominant for current ethanol production based on starch and sugar feedstocks.

To enable cellulosic ethanol technologies, microbial capability and efficiency must be enhanced by appropriately designed mixed‐culture systems and/or genetically modified microbes. Since banana-associated residual biomass are generally starchy (amylaceous) and lignocellulosic materials; they can give high yields of glucose after successful hydrolysis which may further be fermented to produce ethanol. The conversion of starch-based crops such as corn, grains and potatoes, among others, involves the enzymatic breakdown of strong 1,6 glycosidic bonds in starch into simple sugars (glucose) prior fermentation into ethanol [150]. On the other hand, lignocellulosic feedstock such as banana fruit-bunch-stem contains cellulose, hemicellulose and lignin which are more difficult to breakdown than starch and may require concerted efforts involving consortia of microorganism. While one consortium may breakdown the lignin wall, another may be required to hydrolyse the polymer into simpler units for the next consortium. Details of the interplay of these microbial consortia are covered below under the pre-treatment options. Nevertheless, the application of bioethanol fermentation as a waste-to-energy approach has limitations. For instance, conversion of biomass into bioethanol generates other forms of highly polluting wastes such as distillery slope that cannot be directly applied to the fields as biofertiliser or bioslurry. Moreover, the use of bioethanol as engine fuel for generating electricity negatively affects the electric fuel pumps by increasing internal wear and undesirable spark generation. In addition, ethanol is hygroscopic a property that makes it absorb water from air leading to high corrosion progression of energy generating engines and power machines [107].

#### Anaerobic digestion

##### Biochemical and microbial fundamentals of anaerobic digestion (AD)

**Fig. 11:**
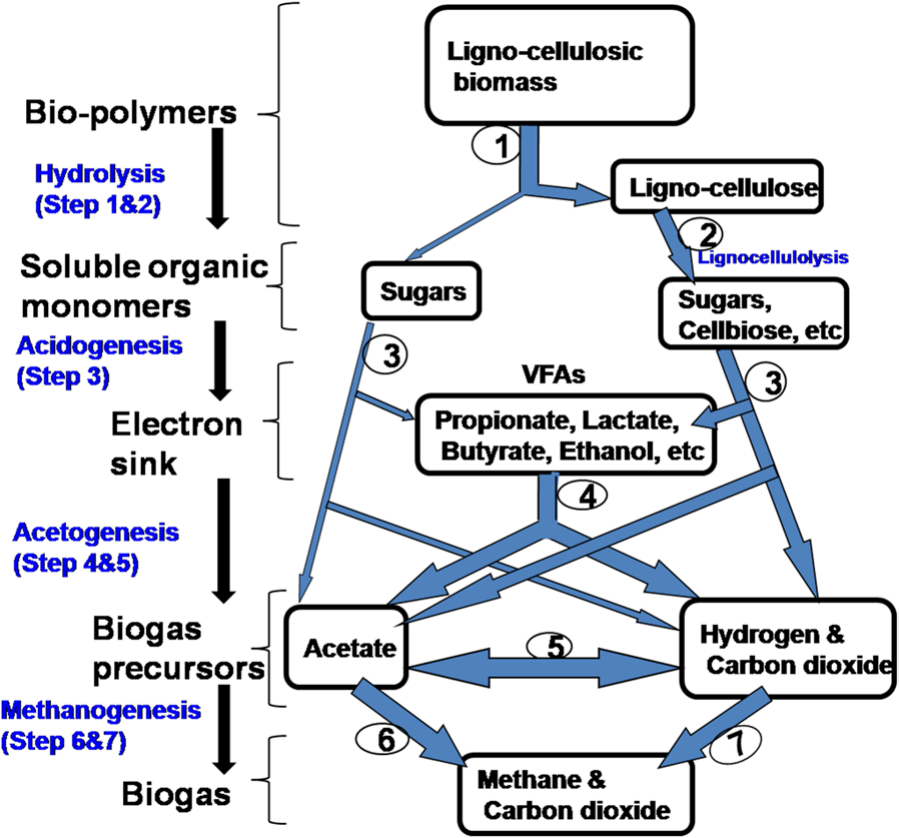
Scheme of anaerobic biodegradation process of lignocellulosic substrate

Anaerobic digestion (AD) is the anoxic biological decomposition of organic matter by a complex microbial ecosystem through parallel sequences of metabolic pathways involving different kinds of synergistic microbial trophic groups leading to the formation of methane and carbon dioxide [66]. The mixture of methane and carbon dioxide is referred to as biogas [42, 43]. Anaerobic digestion offers the opportunity to produce renewable energy and a higher quality of treatment for agro-waste. The technology has recently become an attractive method in Europe for the biodegradation of organic fractions derived from municipal solid waste [145]. The AD process is driven by concerted action of highly varied microbial population, consisting of several groups of both strict and facultative bacterial strains. The process is carried out in well-designed vessel referred to as anaerobic digester/anaerobic bioreactor. The entire system consisting of the feedstock, digester, biogas holder and digestate reservoir is called a biogas plant. The complete AD process of a lignocellulose-rich substrate such as banana waste can be divided into four main stages (Fig. 11) namely: hydrolysis, acidogenesis (or fermentation), acetogenesis and methanogenesis.

- *Stage one: Hydrolysis*

During hydrolysis, the insoluble complex biopolymers such as polysaccharides, proteins and lipids are broken down into simple soluble monomeric biomolecules such as sugars, amino acids, fatty acids and glycerol. It should be noted that organic wastes are a complex mixture of mainly carbohydrates (starch cellulose, hemicellulose), proteins and lipids; with their relative concentrations being dependent on the nature and origin of the waste. Owing to their structural complexity, the biopolymers are not only too large for microbial uptake through the cell membrane for the subsequent intracellular biotransformation steps, they are also either sparingly soluble or completely insoluble in aqueous medium. Therefore, in order to utilise these biopolymeric organics, uptake must hydrolyse them to smaller units and solubilised, to enable membrane uptake and their availability to further metabolic degradation.

Biopolymer hydrolysis is accomplished by means of extracellular hydrolytic enzymes such as laccase, cellulases, amylases, proteases and lipases, which may be either secreted into the environment or secreted but remain bound to cell membrane as protuberances [108, 114, 115, 130]. In the digester system, both mesophilic and thermophilic microbes work synergistically to hydrolyse the biopolymers into simple units (oligomers and monomers). For instance, after the pre-treatment step, the lignin layer would have been removed thereby exposing cellulose, which is a substrate to a number of bacterial genera in the digester. *Clostridium Acetivibrio*, *Bacteroides*, *Selenomonas* and *Ruminococcus* are some of the most common hydrolytic bacteria in the anaerobic bioreactors [16, 17]. In the rumen, the most similar natural environment to biodigesters, *Ruminococcus albus* and *R. flavefaciens* are the predominant gram-positive, fibre-degrading bacteria, while *Fibrobacter succinogenes* is the most abundant gram-negative [180]. Typically, hydrolytic bacteria adhere to the substrate particles, which subsequently induce the production and secretion of the specific hydrolytic enzymes. Starch is broken down by a mixture of amylolytic enzymes that hydrolyse the α-1,4 and α-1,6 glucosidic bonds of amylose and amylopectin. This enzyme mixture includes α- and β-amylase, which exhibit specificity to α-1,4 glycosidic bonds, and glucoamylase (amyloglucosidase), which exhibit specificity to both the α-1,4 and α-1,6 glucosidic bonds [29, 100]. Starch hydrolysis releases a mixture of sugars; notably maltose and glucose. On the other hand, cellulases which are sub-divided into three main groups namely: endocellulase or endo-β-1,4-d-glucanase, (EC 3.2.1.4), exocellulase or exo-β-glucanase, also called cellobiohydrolase (EC 3.2.1.91) and β-glucosidases (EC3.2.7.21), are also secreted by microorganisms in the digester. The degradation of cellulose is effected by the cooperative action of both endocellulase and exocellulases, whereby, the endocellulases randomly hydrolyse internal glycosidic linkages, which are accompanied by a rapid decrease in polymer length and gradual increase in the reducing sugar concentration, while the exocellulases hydrolyse the oligosaccharides released by the endocellulases to produce cellobiose from a non-reducing end. Completed hydrolysis is achieved when β-glucosidase hydrolyses cellobiose to glucose monomers [75, 102]. The cellulase enzyme system is enclosed in a cellulose-binding multicellulase-containing protein complex called a cellulosome. The cellulosome is responsible for the adherence of the bacterial cell to cellulose and to hydrolyse the cellulose thereafter. It should also be noted that the cellulosome complex retains the ability to bind to and hydrolyse cellulose when present in the extracellular medium as it does when it is cell bound [22, 23]. Similar surface structures exist among different cellulolytic bacteria. Typical examples include: (a) glycocalyses, which have been observed in rumen bacteria, (b) fibrous and membranous structures of *Bacteroides succinogenes* and (c) spherical bodies, vesicular structures, lobes and tubelike appendages, which have been observed in *Ruminococcus albus*. The presence of these structures strongly supports the widely held view that a single enzyme is incapable of extensive solubilisation of complex substrates, but rather, multiple enzyme system that act synergistically are required (113). Microorganisms produce both intracellular and extracellular proteases contemporaneously [71]. As with other classes of enzymes, proteases likewise, play major roles in microbial physiology and as such, their production is highly regulated to suit particular needs. The synthesis of extracellular proteases, for example, is also tightly regulated. Their production has been linked to their participation in physiological activities such as sporulation [138], cell wall turnover and autolysis [157], nutrition and overall protein turnover [105]. Lipases (triacylglycerol acylhydrolase; EC.3.1.1.3) hydrolyse lipids or triacylglycerols to diacylglycerides, monoacylglycerols, fatty acids and glycerol. In comparison, hydrolysis of proteins and lipids is faster [128]. Proteins are generally hydrolysed to amino acids by proteases. Microorganisms that are responsible of this reaction include species of the genera *Bacteroides, Butyrivibrio, Clostridium, Fusobacterium, Selenomonas* and *Streptococcus* [8].

(b) *Stage two: Acidogenesis*

In acidogenesis, soluble monomers: simple sugars, amino acids, glycerol and fatty acids released from the hydrolysis stage, are biodegraded by fermentative organisms and anaerobic oxidisers (β-oxidisers) to produce different organic acids. Representatives of domain Bacteria, especially microbial genera inhabiting the rumen: *Clostridium, Eubacterium* and *Bacteroides,* are largely responsible for acid generation. Fermentative species typical of the rumen include species of *Clostridium* and *R. Albus* [49, 153], while *Streptococcus* sp., *Lactobacillus* sp. and *Propionibacterium* are also fermentative microorganisms associated with the biodigesters, probably originating from the environment. Their degradative products of metabolism include acetate, lactate, ethanol, CO_2_ and H_2_ [81]. On the other hand, the deamination process in the degradation of amino acids also produces ammonia. Microbial fermentation of glucose and 5-carbon atom sugars such as xylose and ribose mainly proceed through Embden–Meyerhof Pathways (EMP), generating pyruvate as an intermediate pathway product. However, the formation of pyruvate depends on the conditions prevailing in the bioreactors and the microbial species present. Pyruvate is a central molecule in terms of biochemical interconversions and can be converted into different compounds such as acetate, propionate, butyrate, formate, lactate, alcohols, ketones and aldehydes [133]. The amino acids originating from protein hydrolysis can be degraded either through fermentation following either stickland reactions or via anaerobic oxidation linked to hydrogen production. The protein biodegradation products are volatile fatty acids (VFAs), ammonia, sulphide, carbon dioxide and hydrogen depending on the amino acid present, microbial diversity and the pathway. Butyrate and valerate are typical products of valine and leucine amino acid biodegradation [33, 109, 125]. The acidogenic microbial population can constitute upto 90% of the total microbial populations present in the anaerobic digesters [134]. These microbes have a short doubling time that makes acidogenesis not regarded as a limiting step in the process of anaerobic digestion.

(c) *Stage three: Acetogenesis*

Acetogenesis is the degradation of reduced fermentation intermediates (electron ‘sink’) from the previous stage, i.e. volatile fatty acids (VFAs) such as propionate and butyrate to acetate, carbon dioxide and hydrogen by obligate hydrogen-producing acetogens (OHPA). This intermediate bioconversion is a crucial process for the successful production of biogas, since these compounds cannot be utilised directly by methanogens. However, the acetogenic reactions (Table 3) are not energetically feasible under standard conditions because the reactions are energy consuming (endothermic; +ve values of Δ*G*). Therefore, a syntrophic microbial interdependency is required for the reactions to proceed.

**Table 3:** Free energy values of key acetogenic and methanogenic reactions of anaerobic digestion (Adapted from [42, 108])

AD step	Reaction	∆*G* ^0^ (kJ mol^−1^)*
Acetogenesis		
Propionate → Acetate	${\text{CH}}_{ 3} {\text{CH}}_{ 2} {\text{COO}}^{ - } + 3 {\text{H}}_{ 2} {\text{O}} \to {\text{CH}}_{ 3} {\text{COO}}^{ - } + {\text{H}}^{ + } + {\text{HCO}}_{3}^{ - } + 3 {\text{H}}_{ 2}$	+76.1
Butyrate → Acetate	CH_3_CH_2_CH_2_COO^−^ + 2H_2_O → 2CH_3_COO^−^ + H^+^ + 2H_2_	+48.1
Ethanol → Acetate	CH_3_CH_2_OH + H_2_O → CH_3_COO^−^ + H^+^ + 2H_2_	+9.6
Lactate → Acetate	${\text{CH3CHOHCOO}}^{ - } + 2 {\text{H}}_{ 2} {\text{O}} \to {\text{CH}}_{ 3} {\text{COO}}^{ - } + {\text{H}}^{ + } + {\text{HCO}}_{3}^{ - } + 2 {\text{H}}_{ 2}$	−4.2
Formate → Acetate	$2 {\text{HCO}}_{3}^{ - } + 4 {\text{H}}_{ 2} {\text{O}} + {\text{H}}^{ + } \to {\text{CH}}_{ 3} {\text{COO}}^{ - } + 4 {\text{H}}_{ 2} {\text{O}}$	−104.6
Methanogenesis		
Acetate → Methane	${\text{CH}}_{ 3} {\text{COO}}^{ - } + {\text{H}}_{ 2} {\text{O}} \to {\text{HCO}}_{3}^{ - } + {\text{CH}}_{ 4}$	−31.0
H_2_/CO_2_ → Methane	4H_2_ + CO_2_ → CH_4_ + 2H_2_O	−131.0
Formate → Methane	${\text{HCO}}_{3}^{ - } + 4 {\text{H}}_{ 2} + {\text{H}}^{ + } \to {\text{CH}}_{ 4} + 3 {\text{H}}_{ 2} {\text{O}}$	−135.6

* Temperature 298 K, pH 7, 1 M for solutes and 1 atm for gases

**Fig. 12:**
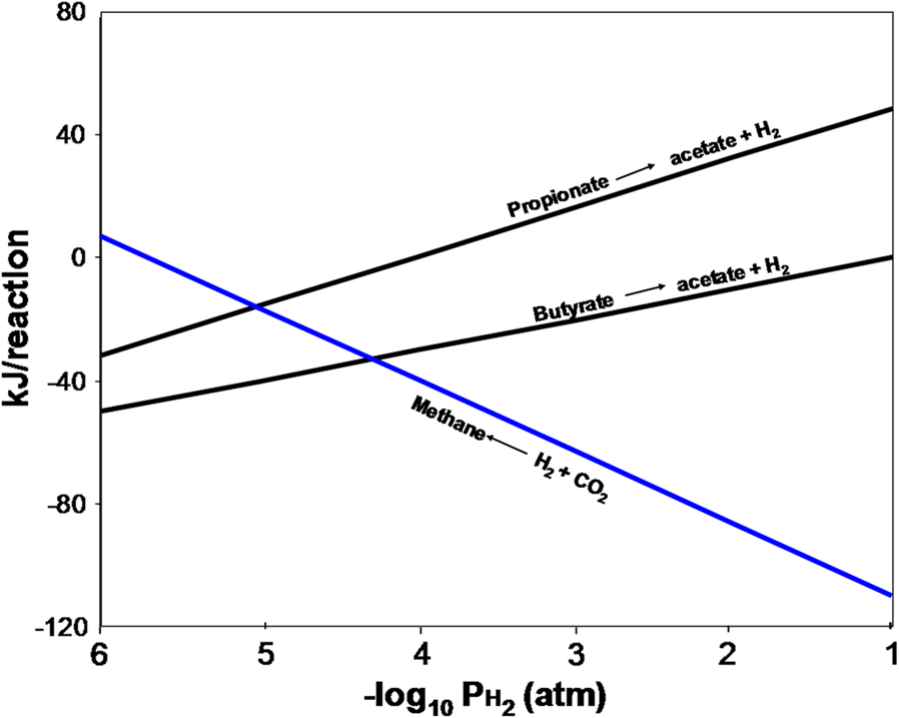
The energetics and effects of hydrogen partial pressure on syntrophic degradation in anaerobic digestion Adapted from [27]

According to Björnsson [27] and Cirne [42], the reactions become feasible when the hydrogen partial pressure (PH_2_) is low (10^−4^–10^−5^ atm). Acetogens are slow-growing microorganisms and depend on a low hydrogen partial pressure in order for acetogenic biodegradation to yield energy required to move the reaction forward [27]. This low (PH_2_) is achieved by the syntrophic association of obligate hydrogen-producing acetogens (OHPAs) with hydrogen-consuming bacteria (hydrogen scavengers) such as the hydrogenotrophic methanogens [147]. However, the thermodynamic feasibility of acetogenic reactions is inversely proportional to that of methanogenic reactions. This means that hydrogen-producing acetogenic reactions become more favourable at low PH_2_ (Fig. 12) whereas hydrogen-consuming methanogenic reactions become less favourable at the same PH_2_. Thus, syntrophic reactions occur within a narrow range of very low PH_2_ (between 10^−4^ and 10^−5^ atm).

Syntrophic acetogenic bacteria include (a) the butyrate-degrading acetogenic bacteria such as *Syntrophomonas wolfei, Syntrophomonas sapovorans* and *Syntrophomonas bryantii*; (b) the propionate-degrading acetogenic bacteria such as *Syntrophobacter wolinii*, *Syntrophobacter phenigii* [42]; (c) the primary alcohol-degrading bacteria encompassing such species as: *Syntrophobacter fumaroxidans, Desulfovibrio vulgaris*, *Thermoanaerobacterium brockii* and *Pelobacter venetianus*; and (d) homoacetogenic bacteria (hydrogen utilising acetogens such as strain AOR) which are responsible for converting acetic acid into hydrogen and carbon dioxide. Acetogenesis is a low energy-yielding anaerobic biodegradation step. This makes acetogenic microbes very slow growing and sensitive to changes in organic loads, flow rate and environmental conditions [186]. Acetogenic bacteria, therefore, require long periods to adapt to new environmental conditions in order to optimise acetogenesis in the bioreactor.

(d) *Stage four: Methanogenesis*

Methanogenesis is the biomethanisation step in which organic substrates: acetate, H_2_/CO_2_, methanol and formate, the end products of the acetogenesis, are converted into methane [65]. Unlike in the previous stages, the microorganisms responsible for the methanogenic stage belong to the domain archaea and they produce methane via two major pathways: acetotrophic (or acetoclastic) and hydrogenotrophic methanogenic pathways (Table 3). It has been estimated from stoichiometric reactions that about 70% of the methane is produced via the acetotrophic pathway [97]. Nevertheless, very few known species can perform acetotrophic methanogenesis, whereas nearly all known methanogenic species are hydrogenotrophic methanogens [27]. Bioenergetically, hydrogenotrophic methanogenic reactions are more favourable (Δ*G* ^0′^ = −131.01 kJ/mol for H_2_/CO_2_ and Δ*G* ^0′^ = −135.6 kJ/mol for H_2_/HCO_3_), while acetoclastic (acetotrophic) methanogenic reactions are least favourable (Δ*G* ^0′^ = −31.0 kJ/mol for CH_3_COOH) as shown in Table 1. The hydrogenotrophic methanogenic pathway is more energy yielding than acetotrophic methanogenic pathway and is normally not rate limiting but rather fundamentally important in keeping the PH_2_ low in bioreactor system, allowing syntrophic acetogenesis to proceed. Hydrogen is recognised as the controlling parameter in the overall scheme of waste biodegradation but rarely detected in well-functioning methanogenic biodigesters [14, 27]. Unlike the acetoclastic methanogens, the hydrogenotrophic methanogens are among the fastest-growing organisms in the anaerobic biodegradation process and the accumulation of hydrogen may only occur during process overloads or toxic microbial inhibition. The minimum doubling time for the hydrogenotrophic methanogens has been estimated to be 6 h compared to 62.4 h (2.6 days) for the slow-growing acetoclastic methanogens [27]. Furthermore, hydrogenotrophic methanogens are more resistant to environmental changes while acetoclastic methanogens are more sensitive which makes their reactions more rate limiting in several cases of anaerobic digestion of organic wastes [27]. The genera *Methanosaeta* and *Methanosarcina* are the only two groups known to carry out the acetotrophic methanogenesis [61]. The microorganisms of the genus *Methanosaeta* have a lower maximum growth rate than those belonging to the genus *Methanosarcina* hence the former dominates the bioreactor at high acetate concentrations and the latter at low acetate concentrations. Other methanogenic groups include methylotrophic methanogens, which utilise methane-containing compounds such as methanol, methylamine and dimethylsulphides [52].

##### Products from anaerobic digestion

**Fig. 13:**
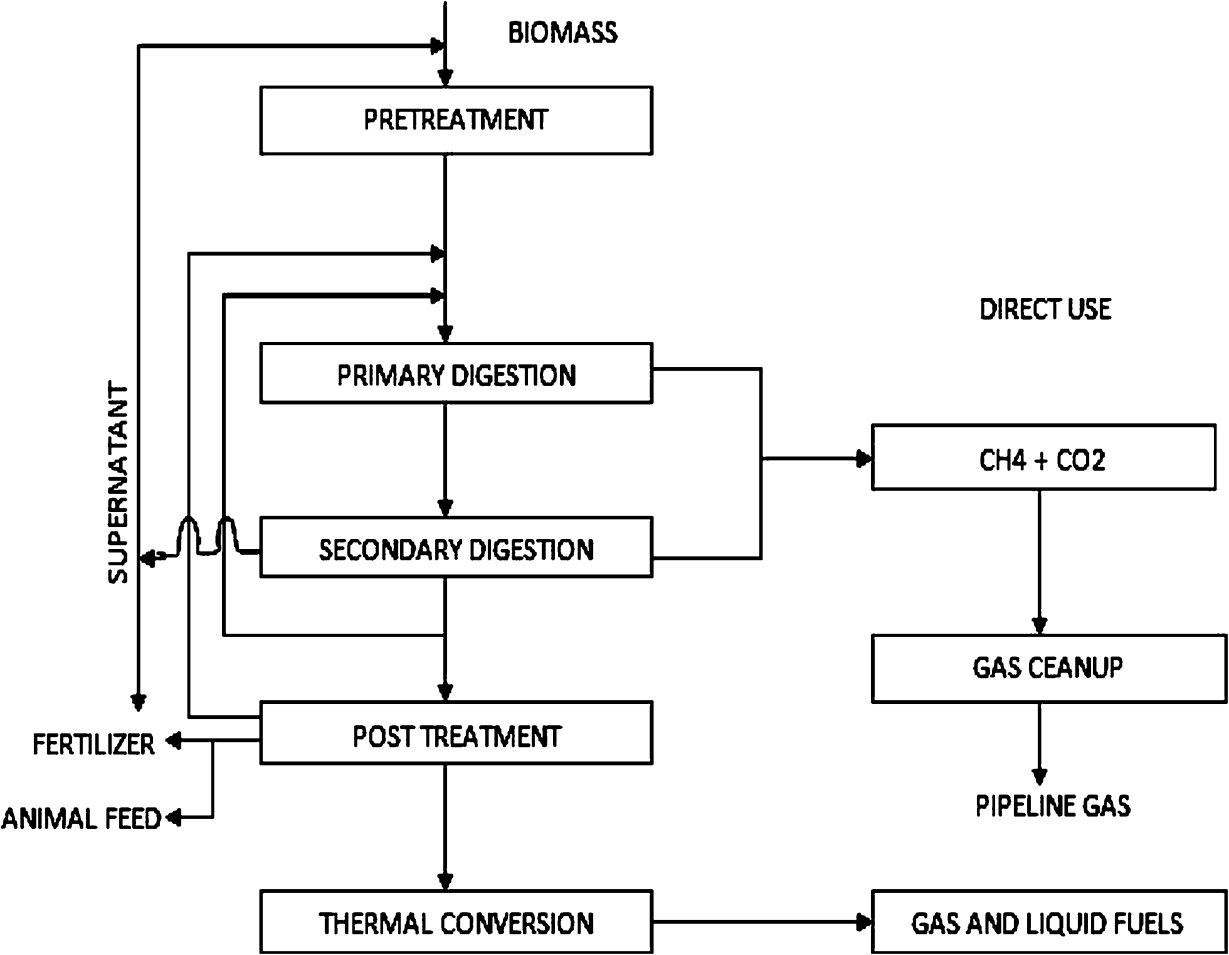
Generalised scheme of major products from anaerobic digestion [163]

In AD, organic waste is fed to the process as feedstock and acted upon by microorganisms in the absence of oxygen [9, 53, 79, 80] to produce biogas and bioslurry. The digestate (bioslurry) can be dewatered and converted through thermal conversion technologies into other forms of fuel including refuse-derived fuel (Fig. 13). The remaining inorganic and the inert waste are either incinerated or gasified to generate more energy. Apart from energy generation, the bioslurry can safely be used as biofertiliser in agricultural production as well as animal feed especially for piggery, fisheries and aquaculture. This makes anaerobic digestion as one of the best waste-to-energy technologies with superior advantage of coupling energy generation with the generation of valuable bi-products such as plant organic fertiliser (bioslurry) at minimal net operational energy requirement. Furthermore, a study by Tock et al. [163] reported that AD is usually a preferred WtE technology for biomass with high water content (including banana waste). It is a low-temperature process that can process wet or dry feeds (with added water) economically at a variety of scales. Results from previous studies on AD of banana peels [44] suggest the high potential and suitability of banana waste as a feedstock for economically viable waste treatment technology like anaerobic digestion for the purpose of energy generation in the form of methane [163]. The composition of the gas produced is primarily carbon dioxide and methane with small traces of hydrogen sulphide.

Besides, the AD of banana waste also reduces global warming and air pollution since the methane produced is considered a clean gas with a zero carbon cycle. Notably, the banana biogas has been proven as a perfectly feasible option to run tractors, farm machinery and vehicles [26], thus offsetting the industrial energy needs. Other advantages of AD process are: reduction in wastes’ pathogens, smaller land suitability and decrease in waste’s pollution potential to levels that are non-toxic to the environment [113].

##### Challenges of using lignocellulosic biomass as feedstocks for anaerobic digestion

Anaerobic digestion of plant biomass as digester feedstocks can be limited by three typical challenges, namely: limited microbial hydrolysis of lignocellulosic biomass, floatation of feed slurry, as well as unbalanced C:N ratio. Limited microbial hydrolysis is one of the major hindrances to AD of lignocellulosic plant biomass such as banana waste, whereby, as much as 50% of the feed substrate could be left undigested.

Lignocellulosic substrates are complex polymeric substances that are insoluble and too large to be taken up by microbial cells for the subsequent intracellular anaerobic degradation steps. Moreover, lignin degradation is primarily an aerobic process, and in an anaerobic environment lignin can persist for very long periods [176]. Therefore to use these lignocellulosic biopolymers as substrates for anaerobic digestion, they must undergo prior solubilisation under aerobic environment. Since biogas digesters are anaerobic, lignocellulosic feedstocks have to first be degraded through pre-treatment stages such as biological hydrolysis under aerobic conditions prior to anaerobic digestion. A research by Mshandete et al. [119] reported that lignocellulosic-rich wastes such as solid sisal residues have high suitability as feedstock for biogas production, after effective hydrolysis. The microbial hydrolysis of lignocellulosic biomass involves several steps, including enzyme production, diffusion, adsorption, reaction and enzyme deactivation step [20]. Hydrolytic enzymes include laccase, cellulase, xylanase and amylase for degrading lignin, cellulose, xylan and starch into oligosaccharides and simple sugars; protease for degrading protein into amino acids, and lipase for degrading lipid into glycerol and long-chain fatty acids [130]. The overall hydrolysis rate depends on organic material size, shape, surface area, enzyme production and adsorption [21]. Moreover, competitive adsorption of enzyme on the inert substrate like lignin can also decrease hydrolysis efficiency [46]. Hydrolysis has been shown to be a rate-limiting step for the digestion of high particulate substrate like agro-industrial residues, municipal solid wastes, swine waste, cattle manure and sewage sludge while methanogenesis is the rate-limiting step for readily degradable substrate, due to the inherent slow growth nature of methanogens (see later) [28].

Floatation of feed slurry in bioreactors digesting the plant biomass is another challenge limiting the use of lignocellulosic material as feedstocks for biogas production. The anaerobic digestion of biomass from plant origin in conventional reactors including the high-rate reactors is generally nuisance and problematic due to the physical nature of the biomass, since these fibre-rich plant biomass materials tend to build up a persistent float layer. The floatation of the feed substrate leads to wash out of active biomass (inocula seeding) that results in digester failure. When feed substrates are discharged early from the reactor, the active flora adsorbed on to the biocarrier gets lost as well, further reducing the efficiency [63]. This has limited the application of high-rate digesters such as upflow anaerobic sludge blanket (UASB) and expanded granular sludge bed (EGSB) reactors, in the treatment of buoyant waste biomass from plant origin and lipid-rich wastes such as fish processing and slaughter house effluents [34, 77, 134]). In order to prevent flotation, intensified agitation and stirring have been recommended and this can demand up to 10% of the electric energy produced after the conversion of the produced biogas into electricity. Intensive mixing can also negatively affect the substrate decomposition process by inhibiting microbial flocculation and adsorption apart from taking up a considerable amount of energy that makes the system economically unattractive. Generally typical biogas digesters in use today cannot efficiently digest lignocellulosic biomass from plant origin such as energy crops without modifications [*Leibniz Institute for Agricultural Engineering Potsdam*-*Bornim* (*ATB*)]. Other research studies reported that AD can proceed at high rate when carried out in appropriately designed bioreactor system with fully optimised environmental and operational parameters [25, 121].

In addition, unbalanced C:N ratio is the other typical challenge faced during anaerobic digestion of lignocellulosic feedstocks from plant biomass. Hydrolysis of lignocellulosic plant biomass mainly releases a lot of sugars comprising simple sugars and oligomers such as multitrioses, with limited nitrogen-rich biomolecules such as amino acids. This implies that there is a high C:N ratio in lignocellulosic plant biomass which can lead to acidic and inhibitory growth conditions for methanogenic bacteria in anaerobic digesters. Successful hydrolysis of lignocellulosic feedstocks such as banana waste can yield a lot of sugars which if converted into organic acids by the acidogenic bacteria, results into bioreactor acidification and inhibition of methanogenesis step. Therefore, before one uses lignocellulosic biomass such as banana waste as a feedstock for biogas production, such apparent challenges ought to be overcome.

##### Options for enhancement of AD of lignocellulosic feedstock

The AD process is influenced by a number of factors leading to varying rates of methane production from a feedstock. The total methane yield and the rate of production, which are a measure of the degree of feedstock microbial digestion, is affected by factors namely: physical–chemical composition of feedstock (feedstock particulate nature), C:N ratio, operating temperature, retention time, inhibitors, agitation (rate of stirring), loading rate and bioreactor configuration. Hence, the AD of plant biomass feedstock such as banana waste can be enhanced through the optimisation of: (a) feedstock pre-treatment, (b) C:N ratio by co-digestion; (c) bioreactor design; and (d) environmental and operational parameters.

- *Feedstock pre*-*treatment*

Pre-treatment is generally feedstock deformation to increase its ability for hydrolysis and absorption by living cells. For lignocellulosic feedstock, an ideal pre-treatment method would increase surface area and reduce lignin content and crystallinity of cellulose [57]. Lignocellulosic biopolymer pre-treatment can be divided into three categories (Table 4) namely: (a) physical methods such as mechanical (milling and grinding), irradiation, steam explosion and hydrothermolysis; (b) thermo-chemical methods (treatment with alkali, dilute acid, oxidising agents, organic solvents and wet oxidation); and (c) biological methods such as whole microbial pre-treatment, enzymatic hydrolysis and bioaugmentation ([119, 120]; Björnsson et al. 2005). Physical/mechanical and chemical pre-treatment methods have been quite intensively studied with the aim of improving the hydrolysis of lignocellulosic substrates. However, these methods have the disadvantages of being either energy intensive or costly and resulting into residual disposal problems [159]. Nevertheless, many researchers have reported that feedstock particle size directly affects the performance of anaerobic bioreactor operating on solid wastes, especially those with a high fibre content [129, 144, 166, 187]. The mechanical size reduction of the particles and the resulting increase in the available surface area represent an option for increasing biodegradation yields and accelerating the AD of substrates that have high fibre content such as banana waste, sisal fibres and straw [11, 68, 119]. A research study by Mshandete et al. [119] demonstrated that feedstocks with high content of fibres such as hay, seeds and leaves give improved digester gas production after mechanical pre-treatment. This leads to a decrease in the amount of residues to be disposed of, and to an increase in quantity of useful digester gas. Therefore it is imperative to pulverise fibrous feedstocks prior to other pre-treatment methods and subsequently anaerobic digestion.

**Table 4:** Some common pre-treatment methods for lignocellulosic biomass (Adapted from [7, 91, 106, 160, 191])

Pre-treatment method	Advantages	Disadvantages
Physical		
Mechanical: Physical reduction in substrate particle size by grinding, milling, etc.	Reduced cellulose crystallinity and degree of polymerization	Usually negative energy balance
	Increased surface area	
Irradiation: Biomass undergoes high-energy radiation (i.e. γ-ray, ultrasound, electron beam, pulsed electrical field, UV, microwave heating)	Results in one or more changes to biomass	Slow
	Increased surface area	Energy intensive
	Reduced cellulose crystallinity and polymerization	Prohibitively expensive
	Partial depolymerization of lignin	
Steam explosion: Substrate particles rapidly heated by high-pressure saturated stream. Explosive decompression caused by quick release of pressure acids released aid in hemicellulose hydrolysis	Causes hemicellulose solubilization and lignin transformation	Destruction of a portion of the xylan fraction
	Cost-effective	Generation of toxin compounds
Hydrothermal: Substrate is subject to high-temperature/high-pressure water	Hemicellulose solubilization	High water and energy demand
	Partial delignification	
Chemical		
Alkaline: Addition of base causes swelling, increasing internal surface of cellulose which provokes lignin structure disruption (NaOH, KOH, Lime, Mg(OH)_2_, NH_4_OH)	Lignin solubilization	Relatively long residence times required
	Reduced cellulose crystallinity and degree of polymerization	Irrecoverable salts formed and incorporated into biomass
	Increased surface area	
	Can be done at ambient temperature	
	Relatively inexpensive	
Acid: Addition of dilute or concentrated acid solutions result in hemicellulose hydrolysis (H_2_SO_4_, HCl, HNO_3_, H_3_PO_4_)	Hemicellulose hydrolysis and converted to fermentable sugars	Relatively expensive
	Alters lignin structure	Corrosive
	With high acid concentration can be done at room temp.	High operational and maintenance costs
		Some inhibitory compounds formed
Catalysed stream explosion: Similar to steam explosion with addition of acid catalyst (SO_2_, H_2_SO_4_, CO_2_, oxalic acid)	Hemicellulose solubilization	Some inhibitory compounds formed
		Portion of xylan fraction lost
		Incomplete disruption of lignin-carbohydrate matrix
Ammonia fibre explosion (AFEX): Substrate is exposed to hot liquid ammonia under high pressure. Pressure is released suddenly breaking open biomass structure	Delignification	Hemicellulose not significantly removed
	Increases surface area	Very high-pressure requirements
	Reduced cellulose crystallinity	Expensive
	Low formation of inhibitors	
Wet oxidation: Dissolved oxygen oxidises substrate	Efficient removal of lignin	High cost of oxygen and alkaline catalyst
	Low formation of inhibitors	High temps and pressures
	Exothermic	
Organo-solvent extraction: Organic solvents are applied, with or without addition of an acid or alkali catalyst to degrade internal lignin and hemicelluloses bonds	Delignification	Solvent removal is necessary
	Some hemicellulose solubilization	Relatively expensive
	Recovery of relatively pure lignin as by-product	
Biological		
Fungi and actinomycetes: Microorganisms degrade/alter biomass structure (white-, brown-, soft-rot fungi )	Degrades lignin and hemicellulose	Low rate of hydrolysis
	Low energy consumption	

On the other hand, biological pre-treatment methods have been reported to be cost-effective and the methods employed are usually simple and involve mild conditions [111]. Biological pre-treatment includes pre-composting and feedstock pre-hydrolysis by either hydrolytic enzymes or pre-culture with hydrolytic enzyme-producing microorganisms [175]. These strategies involve the utilisation of specific microorganisms and/or microbial-derived materials (enzymes) as a means of improving a specific step in the AD process that limits the process. Based on operational approach, the biological strategies include addition of microorganisms or enzymes prior to AD process ([41, 173]; Jeganathan et al. 2007). Others include addition of enzymes directly into the reactor in either a free or an immobilised form [42, 87] and bioaugmentation where specific microorganisms are introduced directly into the digester [43]. Microorganisms, which are naturally growing in lignocellulose-rich waste and other phytomass-rich dumping site, get adapted to degrade lignocellulose waste. A number of microorganisms with the potential for lignocellulose hydrolysis have been previously isolated from such environment and characterised. They include the white-rot fungi of the genera *Phanerochaete, Lentinus and Trametes* Wu et al. [185] and *pleurotus* [132], and bacterial cellulase producers from the *Bacillus subtilis* [95]. Nevertheless, the only organisms known to extensively degrade lignin are fungi [92]. Notably, white-rot fungi are the only known living microorganism capable of complete lignin degradation, and their application has been suggested for delignification of lignocellulosic substrates such as wheat straw [122] prior to AD. The initial reactions are mediated by extracellular lignin and manganese peroxidases, primarily produced by white-rot fungi [92]. Actinomycetes can also decompose lignin, but typically degrade less than 20 % of the total lignin present [18, 47]. Because lignin is an insoluble polymer, the initial steps in its biodegradation must be extracellular. Many enzymes are involved in the oxidative degradation of lignin, including lignin peroxidases (LiP), manganese peroxidase (MnP) and laccase [158].

(b) *Substrate co*-*digestion*

Co-digestion is the anaerobic treatment of a mixture of at least two different nutrient-complementary substrates or waste types. Co-digestion can overcome carbon or nitrogen deficiencies [182]. The mixing of several waste types has a positive synergy on both the AD process itself and on economy of the treatment [78]. Abundance of nitrogen in the substrate can lead to excessive ammonia formation leading to ammonia toxicity and AD process inhibition. Conversely, too little nitrogen creates a risk of nutrient limitation and low buffering capacity incapable to neutralise the volatile fatty acids produced by fermentative bacteria, ultimately resulting in a more pH-sensitive and inhibited AD process [121]. During AD, the microbial community utilises carbon 25–30 times faster than nitrogen [187]. Since not all the carbon and nitrogen in the substrate are available for digestion, the actual C:N ratio is a function of the substrate characteristics and digestion operational parameters. Substrates high in nitrogen can be combined with substrates high in carbon in order to attain the desired C:N ratio for optimal AD process. In general, a C/N ratio of 20–32 has been reported to be the optimal for anaerobic digestion [31, 38, 166, 189]. Furthermore, co-digestion enables the treatment of organic waste with high methane yield due to positive synergies established in the bioreactor [70, 124]. Therefore a suitable ratio of biodegradable carbon to nitrogen can be maintained by co-digestion for efficient AD process. Highly lignocellulosic feedstocks such as wood dust, cotton residues, among others which are rich in carbon but poor in nitrogen should be co-digested with those rich in nitrogen but poor in carbon such as chicken droppings, pig slurry among others. Despite the benefits of co-digestion, co-digestion of mixtures of different wastes including banana waste is seldom reported [48].

(c) *Appropriate bioreactor design*

An anaerobic bioreactor or biogas digester is an enclosed chamber that uses microorganisms to degrade organic matter with the production of biogas. Most farm-based biogas digesters are generally designed for the fermentation of liquid manure and include the traditional floating dome Indian digesters, fixed dome Chinese digester and tubular type. Although these digester types are commonly used in domestic biogas generation, they are associated with significant gas leaks, mainly methane and such defects mainly arise from technical and inappropriate designs which ultimately compromise the efficiency and overall economic value of the digester [72]. This indicates that they are not appropriate for industrial application in the current form and may either be modified or new designs may be made for large-scale industrial applications. Similarly, the high-rate and hybrid digesters that have been modified from conventional digesters to improve anaerobic digestion by sustaining inoculum-substrate exposure and sludge retention are inappropriate for AD of plant biomass and only best suitable for liquid wastes such as waste water effluents. These bioreactors include upflow anaerobic sludge blanket (UASB) and expanded granular sludge bed (EGSB) reactors. When anaerobic digestion of plant biomass is carried out in these conventional bioreactors, the feed substrate slurry tends to build up a persistent float layer that results into discharge of effluent slurry containing partially digested feed substrate and wash out of active biomass (inocula seeding) and ultimately causing AD process failure. Therefore, the efficient anaerobic digestion of lignocellulosic biomass with enhanced biogas production rates requires an appropriate digester design that can circumvent the above heighted challenge.

Biogas digester design must address three major considerations, namely: physical nature and solid content of feedstock, operating configuration mode and bioreactor accessory devices. These factors need to be considered interdependently when designing a bioreactor. The physical nature of feedstocks for anaerobic digestion can be categorised as either solid feedstocks such as fibrous (lignocellulosic) plant biomass, animal tissues (from rendering plants) or liquid feedstock such as high strength wastewaters and sludge. These physical characteristics dictate the design of bioreactor to be used for anaerobic digestion with less complications and optimal biogas production. Generally, feedstocks with less than 15% solid content are termed as wet-pumpable substrates and are appropriately digested by wet bioreactors. On the other hand, feedstock with a solid content of over 25% is termed as dry—stackable substrate and is appropriately digested by dry bioreactors. Bioreactors can be designed, engineered and configured to operate in either batch or continuous process mode. In a batch system, biomass is added to the bioreactor at the start of the process and then sealed for the duration of the process. All the four anaerobic digestion stages occur in one chamber. Batch bioreactors are feasible for highly malodorous and infectious feedstocks such as hospital wastewaters. Constant production of biogas is achieved using more than one batch reactor in series and consequently requires a lot of space. In continuous digestion process mode, organic matter is simultaneously added as the digested material is being removed usually by an automated system. Examples of this form of anaerobic digestion include continuous stirred-tank reactors, upflow anaerobic sludge blankets, expanded granular sludge beds and internal circulation reactors. Such bioreactors are appropriate for liquid slurry such as wastewaters and have constant biogas production. Thick slurry with high solid content (between 15 and 25%) can be digested by wet bioreactors with more energy input to pump the substrate during feeding and slurry removal. The thickness of the material may also lead to bioreactor abrasion and clogging of pipes. On the other hand, dry bioreactors are designed to digest solid substrates of solid content between 25 and 40% without the addition of water, in a process termed as solid-state anaerobic digestion. The primary styles of dry bioreactors are continuous vertical plug flow and batch tunnel horizontal dry bioreactors. Continuous vertical plug flow dry bioreactors are upright, cylindrical tanks where feedstock is continuously fed into the top of the digester, and flows downward by gravity during digestion. In batch tunnel dry bioreactor, the feedstock is deposited in tunnel-like chambers with a gas-tight door. Another design consideration is the necessary accessory device to be fitted with the bioreactor for optimal operation. This consideration is majorly linked with the physical nature of the feedstock to be digested. These devices include feed macerator to reduce particle size and increase surface area for microbial attachment degradation; mixer to re-circulate the feed with microorganism as well as foam reduction; foam controller to disintegrate foam header on the surface of bioreactor liquor; and grit remover to trap sand and other indigestible material from entering the bioreactor.

**Fig. 14:**
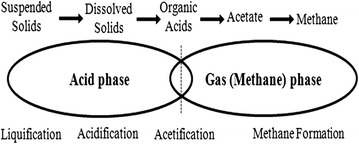
Phase separation of anaerobic digestion system. Adapted from [15]

Besides, the anaerobic digestion (AD) of feedstock in single-phase bioreactors, where all the four stages of AD process occur in one unpartitioned chamber, is always prone to upsets due to contrasting optimal conditions required for both acid and methane formation. The hydrolytic and acid-forming bacteria differ from the methane-forming bacteria in terms of their nutritional needs, growth kinetics and sensitivity to environmental (bioreactor liquor) conditions such as pH. In conventional single-phase bioreactor, the system operates in a narrow delicate balance between acid phase and methane phase (Fig. 14) that must be maintained within the reactor in order to in avoid system failure due to acidification. After successful pre-treatment, the hydrolysis stage of lignocellulosic feedstocks such as banana waste can yield a lot of sugars that when converted to organic acids by the acidogenic bacteria can result into bioreactor acidification and failure. These problems can be circumvented by carrying out a two-phase anaerobic digestion. In the two-phase anaerobic digestion, the process is physically separated into two reactors which offer a method for optimising the operating conditions for the various groups of microorganisms involved in the digestion process. In the two-phase system the first reactor, referred to as the acid-phase reactor is operated under optimal conditions for hydrolysis and acidogenesis while the second reactor is operated under optimal conditions for methanogenesis and is referred to as the methane-phase reactor. In this case, pH and temperature conditions can be maintained at appropriate levels in either reactor. Two-phase digestion can also increase process stability by optimising the hydraulic retention time (HRT) for either phase of the process. Typically, HRT is shorter in the acid phase and longer in the methane phase to accommodate for the variation in growth rate between the rapidly regenerating acidogens and slow-growing methanogens. This can help prevent organic overloading or toxic acid buildup in the methane phase [51].

Ultimately, two-phase operation allows for the selection and enrichment of different bacteria in each phase. Previous research has shown that two-phase anaerobic digestion can be successful in treating lignocellulosic substrates such as forest residues [73] and wood hydrolysate [36]. A report by Zhang [190] also revealed that the acetate-utilising methanogens was 2–10 times higher in the two-phase system than in the single-phase system. Therefore a well-designed two-phase bioreactor system can circumvent the problems associated with bioreactor acidification and enhance the AD process leading to high methane yields.

(d) *Optimisation of environmental and operational parameters*

Environmental parameters are conditions that can be routinely modulated (optimised) either manually or automatically to create suitable environment for microorganisms and consequently enhancing the anaerobic digestion process [42, 60]. These environmental conditions include volatile fatty acids (VFAs), pH, temperature, alkalinity, microbial granulation and their optimal levels (Table 5) are closely affected by the operational parameters. The operational parameters include Organic loading rates (OLR), agitation/stirring, hydraulic retention time (HRT), biomass retention and effluent recirculation among others. Disturbances in reactor equilibrium can result in process inhibition and possible reactor failure.

**Table 5:** Optimal environmental parameters for a stable anaerobic digestion

Environment parameter	Stage of anaerobic digestion process	Optimal range	References
pH	Hydrolysis and acidogenesis (two-phrase anaerobic digestion)	5.5–6.5	[91]
	Methanogenesis (two-phase anaerobic digestion)	6.5–8.5	[15, 91]
	Mixed reactor liquid (one-phase anaerobic digestion)	6.7–7.8	[27, 42]
(PH_2_) (Hydrogen Partial pressure)	Mixed reactor liquid (one-phase anaerobic digestion)	10^−4^–10^−5^ atm	[27, 42]
Alkalinity	Mixed reactor liquid (one-phase anaerobic digestion)	1200–2300 mg CaCO_3_ per litre	[118]
C:N ratio	Mixed reactor liquid (one-phase anaerobic digestion)	20–30	[15, 38]
NH_3_-Nitrogen	Mixed reactor liquid (one-phase anaerobic digestion)	50–200 mg per litre	[118]
Free NH_3_	Mixed reactor liquid (one-phase anaerobic digestion)	<150 mg per litre	[118]
H_2_S	Mixed reactor liquid (one-phase anaerobic digestion)	<200 mg per litre	[54]
Heavy metals	Mixed reactor liquid (one-phase anaerobic digestion)	<10^−4^ M	[27]

(i) * Retention time (RT)*

In anaerobic digestion, retention time is defined as the average time spent by the substrate inside the digester before it comes out after the action of microorganisms in the bioreactor. Retention time is one the key factors that controls the extent to which volatile solids in the substrate are converted to biogas. In typical continuous stirred-tank anaerobic digestion systems the solids retention time (SRT) is equal to the hydraulic retention time (HRT). HRT is directly related to reactor volume, by the equation:

$${\text{HRT = (}}V ) / (Q ),$$

where *V* is reactor volume and *Q* is influent flow rate

Short HRT results into faster wash out of active biomass than they can reproduce, consequently causing prolonged lag phase of some steps such as fermentative step [60]. However, shorter retention times are preferred for waste treatment in order to reduce system costs and increase process efficiency. Shorter HRT is achieved at higher anaerobic digestion rate that is mainly influenced by substrate characteristics. Substrates containing high amounts of lignocellulose require relatively long HRTs in the range of 60–90 days in order to achieve nearly complete digestion of lignocellulosic substrates [141]. AD carried out in conventional bioreactor requires sufficient volume to give long retention time enough for efficient and effective biodegradation of organics. However, too long HRT requires large volume of the digesters that are limited by cost, treatment capacity, net energy yield and operational skills. Conventional anaerobic digestion processes operate at an HRT in the optimal range of 15–30 days [103]. For continuous waste-generating industrial processing, an HRT of 15 days would be optimally ideal although it may be practically impossible for AD of lignocellulosic waste without pre-treatment.

In addition to substrate characteristics, short HRT is also limited by microbial regeneration rates. Methanogens are relatively slow growers and require at least 10–15 days of retention in order to regenerate. Due to this slow regeneration time of methanogens, reactor startup require longer HRTs in order to allow enough time for inoculum sludge to reach a steady-state population [38]. Limitation of slow microbial regeneration rates can also be overcome by appropriate reactor design containing microbial attachment biocarriers and membrane filters that retain microbial biomass during effluent slurry discharge. However, this might result into sludge buildup leading to bioreactor clogging. Thus typical retention time for biogas units is in the range of 20–60 days [67]. Moreover, optimal HRT may vary from 30–50 days in tropical countries and goes up to 100 days in colder climates [187].

(ii) *Organic loading rate*

Organic loading rate (OLR) is defined as the amount of volatile solids or chemical oxygen demand fed to the system per unit volume per day [106]. There is a balance between OLR and HRT that must be determined in order to optimise digestion efficiency and reactor volume. As a consequence, conventional high-rate reactors digesting energy crops can only handle around 3–4 kg of organic dry matter per cubic meter of working volume and per day [63]. Higher OLR can lead to an inhibition of the AD process due to the buildup of volatile fatty acids. At higher OLRs, retention times must be long enough such that the microorganisms have enough time to sufficiently degrade the material. A study by Kirtane et al. [93] established that bioreactors fed with lignocellulosic biomass such as, fruit residues, banana waste among others at higher OLR of over 3.5 results into decrease in methane yield due to microbial inhibition by tannins, alkaloids, flavonoids and terpenoids originating from degradation of plant cell wall. Nevertheless, higher OLRs can allow for smaller reactor volumes thereby reducing the associated capital cost for waste treatment through anaerobic digestion.

(iii) *Feedstock C:N ratio*

Carbon to nitrogen ratio (C/N) is defined as the relative amounts of elemental carbon and nitrogen present in the substrate [106]. In general, a C/N ratio of 20–30 is considered optimal for anaerobic digestion [38, 189]. Substrates with high C/N ratios, such as paper and most crop residues are usually deficient in nitrogen, which is an essential nutrient for microbial cell growth. Thus, anaerobic digestion of very high C/N ratios such sisal waste, wood dust and banana fruit-stalks may be limited by nitrogen availability. In the case of substrates with low C/N ratios, such as some animal manure, toxic ammonia buildup may become a problem. To overcome deficiencies in either carbon or nitrogen, co-digestion of low C/N ratio substrates with high C/N ratio substrates has been proven as an effective solution [69].

(iv) *Bioreactor liquor mixing*

Mixing of bioreactor contents is an important factor in achieving optimal biodegradation of substrate and enhanced methane yield [60]. The mixing assures that all biodegradable matter (metabolites) comes into contact with the biocatalysts (bacteria or enzymes) and removes products (such as biogas) from the system. Mixing also serves to prevent pronounced temperature gradients within the digester and provides a uniform bacterial population density as well as preventing scum formation and decantation of organic matter. Gentle or slow mixing is necessary to maintain process stability within the reactor [189] and hence improving anaerobic digester performance [39, 178]. However, excessive mixing especially stirring at high rate using mechanical devices can disrupt the anaerobic microorganisms, and therefore consideration must be taken in terms of intensity and duration of mixing. Effective mixing of digester contents can be carried out in a number of ways such as stirring using mechanical devices and flushing nozzles, recirculation of biogas and effluent slurry as well as using a wave of feed influx [177, 187]. Mshandete et al. [118] reported that regular shaking (either manually or automatically by shakers) of batch bioreactors especially at laboratory scale can enhance anaerobic digestion. Other related studies have revealed that optimal mixing can achieved by bioreactor stirring at 60 rpm for 15 min/h [184]. In addition to convention bioreactor liquor mixing, liquid recirculation is often adopted for upflow anaerobic sludge blanket (UASB) reactors treating acidic waste such as high carbohydrate wastes to achieve the re-use of the internally generated alkalinity to maintain the pH around neutral in the sludge bed [121]. This leads to reduction in the operational costs of treatment due to savings in alkalinity addition. Furthermore, recirculation of effluent liquor or leachate back to the top of the same bioreactor promotes the dispersion of inoculants, nutrients and acids. The performance of dry batch anaerobic digestion has been reported to be enhanced by leachate recirculation [160]. The same study also reported that the leach-bed bioreactor design uses recirculation of leachate between new and mature bioreactors to inoculate, moisturise and provide nutrients for rapid startup of new bioreactors (fresh waste bed) during anaerobic digestion of solid organic waste. Ultimately, recirculation of leachate removes any buildup of solubilised products, which might otherwise inhibit degradation. The organic acids produced during startup are conveyed to the mature bed where they converted to methane [96].

(v) *pH*

The pH influences the activity of microorganisms and enzymatic activity as they are both active within certain narrow pH ranges [42, 55]. However, due to the formation of different intermediates, pH varies within each phase of anaerobic digestion. At the same time, the different microbial groups involved in each phase require different pH conditions for optimum growth. This stratification of pH along phases of anaerobic digestion affects the growth of certain microorganisms differently. In general, hydrolytic and acidogenic bacteria prefer slightly acidic conditions near pH 6. Optimal pH for acidogens has been reported in the ranges of pH 5.5–6.5 [91] and 5.8–6.2 [192]. In contrast, acidic conditions are toxic to methanogenic bacteria, which prefer neutral conditions in the range of pH 6.5–8.2 [91]. The growth rate of methanogens falls sharply below pH 6.5 [116]. The pH-related inhibition of microorganisms in anaerobic digestion process is caused by reactor imbalances between compounds such as ammonia and volatile fatty acids. As a result, acid accumulation is one of the biggest potentials for anaerobic digester failure. Thus to ensure stable operation in batch bioreactors (one-stage anaerobic digestion process), pH should be maintained between 6.7 and 7.4 [27, 42]. In a properly balanced reactor, pH is buffered through the generation of bicarbonate by methanogens [189]. Providing excess alkalinity through blending of high carbohydrate waste feedstock with alkaline compounds or appropriate substrate co-digestion can buffer the AD process against inhibition due to excess acid accumulation.

(vi) *Temperature*

**Fig. 15:**
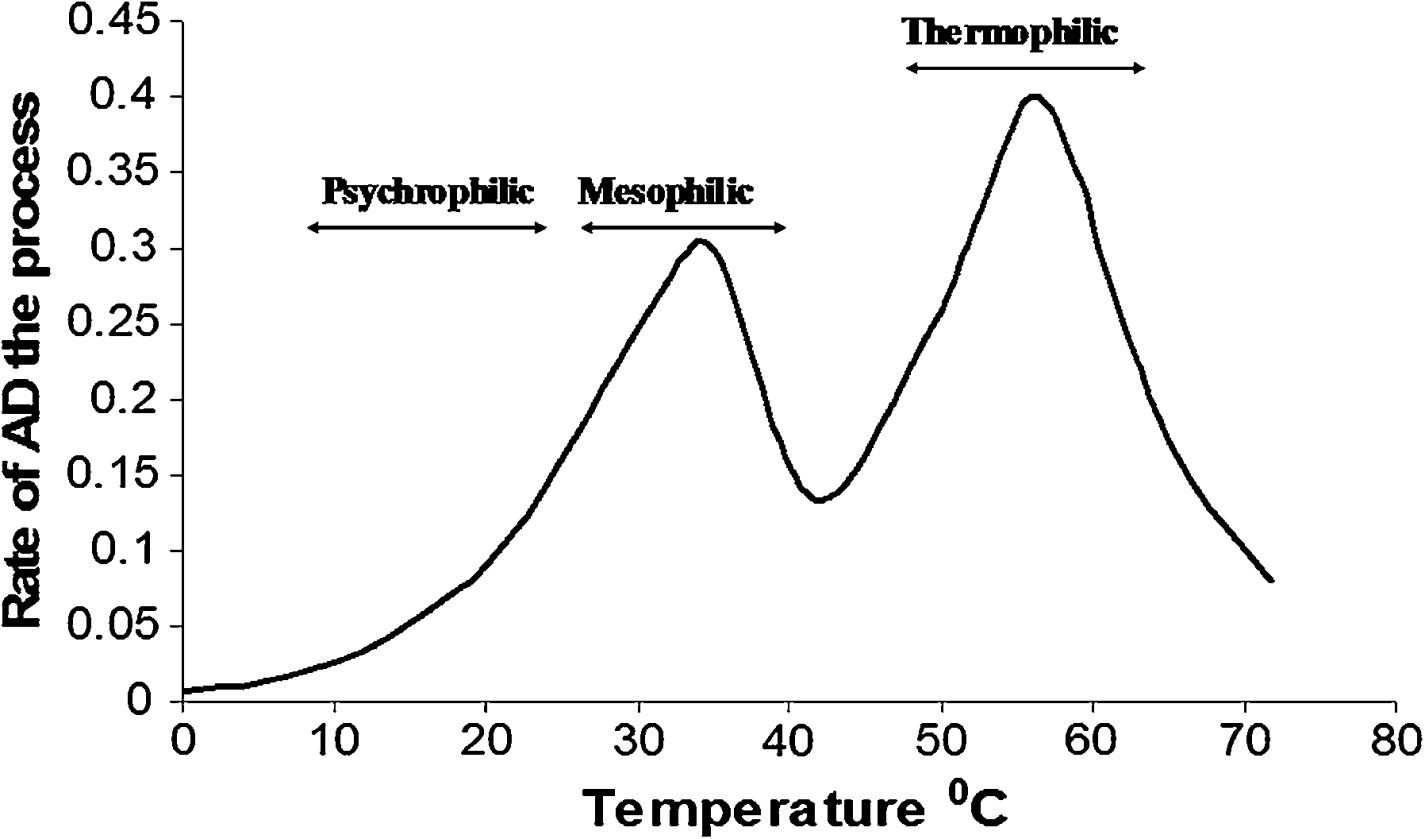
Temperature ranges for anaerobic digestion; optima are 35 °C for mesophilic range and 55 °C for thermophilic range. Adapted from [108]

Microorganisms are divided into three groups depending on their optimal growth temperature: psychrophilic (10–15 °C), mesophilic (30–40 °C) and thermophilic (45–65 °C). Similarly, anaerobic digestion occurs over a large range of temperature (Fig. 15); from psychrophilic temperature at around 10 °C to some extreme thermophilic temperatures over 70 °C [4, 146]. However, anaerobic digesters are usually operated in the mesophilic range with the optimal at 35 °C, or in the moderate thermophilic range with the optimal at 55 °C [108, 175]. Temperature significantly influences anaerobic reactions both from the kinetic and thermodynamic point of view. Hydrolytic and methanogenic biodegradation rates increase with temperature up to certain temperature optima.

In general, higher organic loading rates can be applied in the thermophilic range because of higher microbial growth rate and activity [55]. However, the activity of other groups of bacteria such as propionate and acetate degradation has been shown to decrease when temperature is increased above 60 °C [175]. In addition, the process reactions occurring in the thermophilic range are also more sensitive to toxicity [10, 55]. At higher temperatures, some imbalances can occur such as those resulting from higher acidogenesis (over VFA production) than methanogenesis (low conversion of VFA at higher temperature). Most conventional anaerobic digestion processes occur under mesophilic temperatures due to stability mesophilic conditions that requires less energy input compared to operation under thermophilic conditions, and results in a higher degree of digestion compared to operation under psychrophilic conditions [38, 91]. Within each temperature range, fluctuations in temperature by even a few degrees can affect microbial activity. A study by Chae et al. [35] reported that a fluctuation from 35 to 30 °C caused a significant reduction in biogas production rates. It is therefore important to maintain temperature constant and uniform throughout the digestion process.

## Future trend

This review has indicated that anaerobic digestion is the most appropriate eco-friendly WtE option for the valorisation of banana waste. However, application of this technology to realise high-energy yields in the form of methane requires a lot of modification with the feedstock, bioreactor design and optimisation of operational parameters. Although a number of lignocellulosic pre-treatment methods have been greatly studied, there are still challenges that need further investigation and improvement. Chemical pre-treatment generally leads to residual chemical disposal problems and extra cost for neutralisation of chemical-treated feedstock prior to anaerobic digestion. Hence, further research is needed to focus on microbial pre-treatment especially focusing on development of a viable microbial consortium with efficient lignocellulolytic activity, since lignocellulosic degradation require sequential interplay of different individual microbial strains. Furthermore, the problems associated with plant biomass clogging of conventional high-rate bioreactors and process failure due to feedstock floatation need for more research into development of solid-state anaerobic digesters that are more tailored for biomethanisation of high solid feedstocks such as plant biomass including energy crops and banana waste. Since banana waste has high moisture content, it could be digested without additional water requirement. The design and engineering of a future solid-state digester tailored for anaerobic digestion of plant biomass should ensure that it:

- Operates in a semi-continuous mode to allow sustainable gas production all throughout without interruption like that caused by batch reactors.
- Has mixing devices to mingle incoming (fresh) solid feedstock with the leachate inoculums.
- Re-circulates effluent slurry or leachate back to the digester to re-inoculate the incoming solid feedstock and minimise water usage.

Lastly, further research into standardisation of optimal operational parameters for anaerobic digestion of lignocellulosic feedstocks will be imperative for full-scale application of the technology for industrial and large-scale energy generation.

## Conclusion

In this review, the waste-to-energy technologies that are potentially applicable to Uganda’s banana industrialisation were highlighted. Generally, both thermal and thermo-chemical conversion technologies can positively generate net energy if the processes do not require additional fuel input. Direct thermal and thermo-chemical conversion technologies would be inappropriate Waste-to-Energy options for wastes with high moisture content such as banana waste due to low net energy yield despite their superior potential for complete pathogen destruction. The net energy yield of biomass through thermal conversions is directly related to the moisture content of substrate. Banana waste can be on positive net energy balance through direct thermo-chemical conversions when the substrate had prior drying before thermal degradation. Therefore, thermo-conversion options seem less favoured due to the high moisture content of banana waste. On the other hand, biochemical conversion technologies are more favoured by such moisture content in addition to being more eco-friendly. Among these technologies, anaerobic digestion stands out as the most feasible waste-to-energy technology for Uganda’ banana industrialisation mainly due to limited technical knowledge and economic capability to employ more sophisticated energy conversions such as supercritical water gasification, pyrolysis and bioethanol production. Moreover, anaerobic digestion is a more appropriate waste-to-energy technology for banana waste since the latter is high organic and purely biodegradable with release of carbohydrates especially starch and lignocelluloses that have high net potential for production of energy in the form of biogas. Besides, the effluent digestate waste from anaerobic digestion is a cheap source of nutrient-rich plant biofertiliser which can be re-applied to plantation to boost crop production.

## Abbreviations

BW: banana waste
WtE: waste-to-energy
TOPs: torrefied pellets
ASP: aerated static pile
AD: anaerobic digestion
EC: enzyme code
PH_2_: hydrogen partial pressure
HRT: hydraulic retention time
OLR: organic loading rate
VFA: volatile fatty acids
